# Selective targeting of a histone-like silencer Sfx to the R6K conjugal transfer operon

**DOI:** 10.1093/nar/gkag583

**Published:** 2026-06-10

**Authors:** Bing Wang, Ritika Gupta, Nathan Blaine, Barbare Khitiri, Catherine Jordan, Natalia Molotievskiy, David Dunlap, Laura Finzi, Irina Artsimovitch

**Affiliations:** The Department of Microbiology, The Ohio State University, Columbus, OH 43210, United States; The Center for RNA Biology, The Ohio State University, Columbus, OH 43210, United States; Department of Physics and Astronomy, Clemson University, Clemson, SC 29634, United States; The Department of Microbiology, The Ohio State University, Columbus, OH 43210, United States; Department of Chemical and Biological Science and Engineering, United States Military Academy, West Point, NY 10966, United States; The Department of Microbiology, The Ohio State University, Columbus, OH 43210, United States; Department of Bioengineering, Clemson University, Clemson, SC 29634, United States; The Department of Microbiology, The Ohio State University, Columbus, OH 43210, United States; Department of Physics and Astronomy, Clemson University, Clemson, SC 29634, United States; Department of Physics and Astronomy, Clemson University, Clemson, SC 29634, United States; Department of Bioengineering, Clemson University, Clemson, SC 29634, United States; Medical Biophysics Program, Clemson University, Clemson, SC 29634, United States; Institute for Human Genetics, Clemson University, Clemson, SC 29634, United States; The Department of Microbiology, The Ohio State University, Columbus, OH 43210, United States; The Center for RNA Biology, The Ohio State University, Columbus, OH 43210, United States

## Abstract

Conjugative plasmids drive bacterial evolution and antibiotic resistance spread, yet their gene expression must be silenced to protect the host. A histone-like protein H-NS represses many mobile and sedentary xenogenes but fails to silence the conjugal transfer *vir* operon of R6K, a prototype IncX plasmid. Instead, R6K encodes its own H-NS homolog, Sfx, to repress the *vir* operon. Here, we show that, unlike other plasmid silencers that target promoters, Sfx cooperates with Rho factor to arrest transcription elongation. ChIP-seq reveals that Sfx and H-NS share similar DNA motifs and a preference for negative supercoiling, but occupy reciprocal genomic niches; Sfx is enriched on the R6K *vir* operon despite weak chromosomal binding, whereas H-NS displays the opposite preference. We show that Sfx binding to *vir* DNA critically depends on DNA topology and hypothesize that its selective targeting to R6K is mediated by Sfx-*vir* interactions and phase separation. Our results suggest that Sfx phase separates with R6K to ensure its preferential recruitment to the plasmid DNA and forms stable bridged nucleoprotein filaments that are impermeable to competitors such as H-NS. These findings reveal how histone-like proteins can partition the genome into distinct regulatory niches, a strategy likely mirrored across all life.

## Graphical Abstract

**Graphical Abstract:**
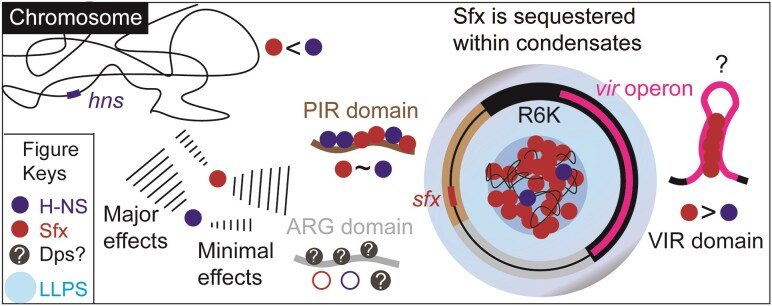

## Introduction

Conjugative plasmids transfer among bacteria, even across different species and genera [1], driving the bacterial evolution and the dissemination of multidrug resistance [2]. Plasmids readily accumulate “beneficial” genes, such as those encoding resistance to antibiotics, toxins, and heavy metals, as well as virulence factors, and share these genes with other mobile genetic elements, facilitating the assembly of new combinations of traits [3].

While horizontal gene transfer enables rapid adaptation to hostile environments, it may come at a cost. Conjugative DNA transfer is mediated by large membrane-spanning type IV secretion system (T4SS) complexes [4] encoded by very long plasmid operons. Expression of the transfer operons (commonly called *tra* or *vir*; see [5] for the current classification) is associated with cellular stress [6] and can impose a metabolic burden [7]. Even more crucially, the conjugative pili serve as receptors for phages [8]. To minimize these risks, the *tra* genes must be tightly regulated.

Many long operons exhibit polarity, a decrease in the levels of RNAs transcribed from the distal genes of the operon. Polarity is imposed by Rho, a transcription termination factor that triggers premature release of RNAs that are not actively translated [9], a common feature of AT-rich xenogenes [10]. Rho-dependent termination is aided by NusG [11, 12] and H-NS, which binds to AT-rich DNA motifs and spreads to form extended nucleoprotein filaments that can hinder RNAP binding to promoters or create blocks to elongation [13]. Together, Rho, NusG, and H-NS silence pathogenicity islands and other xenogenes [14].

RfaH, a sequence-specific paralog of NusG, relieves Rho and H-NS silencing. RfaH abolishes Rho-mediated polarity [15] by inhibiting RNAP pausing [16], excluding NusG from RNAP [17], and activating translation [18]. RfaH also counter-silences H-NS [19, 20], presumably by destabilizing bridged nucleoprotein filaments that are formed by H-NS and accessory proteins (StpA and Hha) to trigger premature termination [21].

**Figure 1.**
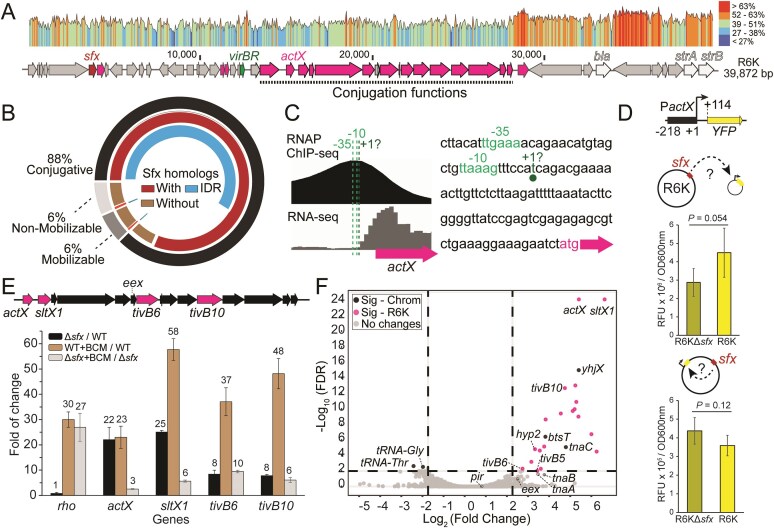
R6K-encoded Sfx represses the plasmid conjugation functions. (**A**) R6K map. The three antibiotic-resistant genes (*bla, strA, strB*) are in light gray; genes that encode predicted positive regulators of conjugation, *virBR* (green) and *actX* (magenta), and the silencer *Sfx* (red) are indicated. The GC content (calculated with Snapgene v8.2.2) is shown above the map. Genes significantly upregulated by the *sfx* deletion are highlighted in magenta (see panel F). (**B**) Sfx is the core gene of IncX plasmids (a total of 1881 IncX plasmids were analyzed). The outer ring shows the distribution of the three mobility groups in IncX plasmids. The middle ring indicates the presence of Sfx homologs. The inner ring shows the presence of intrinsically disordered regions (IDRs) in Sfx homologs. (**C**) RNAP ChIP-seq reveals the location of P*actX* promoter. The −10 and −35 elements are predicted by BPROM, and the transcription start site (+1) is predicted by BDGP. RNA-seq track is smoothed in 20-bp windows. The sequences of −10 and −35 elements are shown on the right. (**D**) Relative fluorescence unit of yellow fluorescent protein (YFP), normalized by OD_600_ nm. The effects of Sfx on P*actX* were tested using the P*actX*-*yfp* reporter located on a P15A plasmid (*in trans*, top) or on R6K (*in cis*, bottom). Error bars are the standard deviation (SD; *n* = 5). A two-tailed *t*-test assuming unequal variances was used to calculate *P* values. (**E**) Reverse transcription quantitative polymerase chain reaction (RT-qPCR) analysis of selected genes. WT, wild-type R6K. BCM, bicyclomycin. Δ*sfx*, R6KΔ*sfx*. Mean values are indicated at the top of the bars. Error bars are SD (*n* = 3). (**F**) Volcano plot of RNA-seq data. The significantly changed genes (FDR < 0.01, ABS(log_2_FC) > 2, FC (Fold Change) = R6KΔ*sfx* / R6K WT) are highlighted in black for chromosomal and magenta for R6K genes.

As plasmids are mobile xenogenes, finding that they are regulated similarly to their sedentary relatives appears hardly surprising. However, some conjugative plasmids encode paralogs of H-NS and RfaH. R6K, the prototype plasmid of the IncX group, is a 40-kb IncX2 plasmid that has been used as a model system for plasmid replication and conjugation studies for decades (Fig. 1A). R6K contains three antibiotic-resistant genes located in a GC-rich transposition island and genes required for plasmid replication, segregation, and transfer (the *vir* operon and relaxosome *taxA/C* genes). R6K also encodes three regulators of conjugation: ActX, a homolog of RfaH; Sfx, a homolog of H-NS, and VirBR (Fig. 1A). VirBR and ActX have been reported to activate conjugation in other IncX plasmids [22, 23], while Sfx silences R6K conjugation [24], but their molecular mechanisms are unknown.

Our bioinformatic analysis (Dataset 1A) shows that 88% of IncX plasmids are predicted to be conjugative (Fig. 1B), and >90% of these encode one or two Sfx homologs. In comparison, only 7%–8% of mobilizable or non-mobilizable plasmids have Sfx. Thus, *sfx* is a core gene in conjugative IncX plasmids that silences conjugation of R6K [24] and other IncX plasmids [22, 23]. A recent phylogenetic analysis reveals that Sfx has diverged from the H-NS paralog StpA, undergoing positive selection in several regions, including the key DNA-binding AT-hook motif [24]. Similar to StpA [25] and Sfh, an H-NS like protein encoded on the IncHI1 plasmid [26, 27], Sfx could partially complement the phenotypes of the *hns* deletion [24]; however, H-NS did not inhibit conjugation of R6KΔ*sfx* [24]. This one-way complementation is particularly striking given that H-NS is known to bind AT-rich DNA, such as the R6K *vir* operon (Fig. 1A), with loose specificity (reviewed in [10]). The inability of H-NS to substitute for Sfx suggests that H-NS either does not bind to the R6K *vir* operon or is unable to silence its expression. However, nothing is known about the molecular details of Sfx binding to chromosomal and plasmid DNA and the mechanism by which Sfx silences R6K conjugation.

Here, we show that Sfx shares many chromosomal targets with H-NS but exhibits an apparently low occupancy. Consistently, the *sfx* deletion does not alter transcription of most associated chromosomal genes. This pattern is reversed on R6K: Sfx binds to and strongly represses the *vir* operon during transcription elongation, acting together with Rho, whereas H-NS binds to the *vir* operon weakly and does not affect R6K conjugation. Our analysis of DNA recognition motifs, target shuffling, and *in vitro* DNA-binding assays show that the selective recruitment of Sfx to R6K DNA is mediated by sequence-specific contacts and is influenced by template topology but is less sensitive to the target location. Finally, we show that Sfx can form condensates with R6K, a property that may favor its preferential binding to the plasmid DNA.

## Materials and methods

Plasmids, oligonucleotides, and strains used in this study are listed in Supplementary Table S1. Restriction and modification enzymes for plasmid construction were obtained from New England Biolabs, DNA—from Millipore Sigma, and synthetic DNA fragments for HiFi assembly—from Genscript. The sequence of all plasmids was confirmed by whole-plasmid sequencing at Plasmidsaurus; the GenBank-format files are included in Dataset 2. Antibiotics were added when needed: carbenicillin (100 μg/ml), kanamycin (40 μg/ml), chloramphenicol (30 μg/ml), and tetracycline (15 μg/ml).

### Sfx homologs distribution in IncX plasmids

IncX plasmid metadata, including NCBI accession ID and predicted mobility, were downloaded from PLSDB (v. 2024_05_31_v2) [28]. Multi-replicon plasmids were excluded from further analysis. A total of 1881 IncX plasmid sequences were downloaded from NCBI. Using the R6K Sfx as a query, a BLASTp search (BLAST + v.2.9.0) [29] was performed with an *E*-value cutoff of 10^−10^ against the downloaded sequences to collect Sfx homologs. In all BLAST searches, truncated homologs that consist solely of an N- or C-terminal domain were excluded from consideration by restricting searches to proteins with >50% coverage of Sfx. The IDRs were predicted by metapredict v3 [30].

### Protein purification

*Escherichia coli* BL21 (DE3) cells were used for protein overexpression. Cells harboring the expression vector (Supplementary Table S1) were cultured in Terrific Broth (Research Products International, cat# T15100) at 37°C, and isopropyl-1-thio-β-D-galactopyranoside (IPTG; Goldbio, cat# I2481C) was added when OD_600_ reached ∼ 0.5. For H-NS and Sfx, 0.5 mM IPTG was used to induce expression for 2.5 h at 37°C. For Hha, 0.1 mM IPTG was used for an overnight induction at 16°C. The cells were collected by centrifugation at 8000  ×  *g* for 8 min at 4°C.

*H-NS and Sfx mutants*: The cell pellet was resuspended in Lysis buffer A [50 mM Tris–HCl, pH 8, 5% (v/v) glycerol, 1 M KCl, 10 mM β-mercaptoethanol (β-ME), 1× ProBlock Gold 2D Protease Inhibitor Cocktail ethylenediaminetetraacetic acid (EDTA) Free; Goldbio, cat# GB-109-1)] and opened by sonication. The cell lysate was cleared by centrifugation (20 000  ×  *g*) for 30 min at 4°C. The supernatant was passed through a 0.45 μm filter and applied to High Affinity Ni-charged Resin (Genscript, cat# L00223). The resin was washed with Ni-A buffer [40 mM Tris–HCl, pH 8, 5% glycerol, 1 M KCl, 10 mM β-ME, 0.2 mM phenylmethylsulfonyl fluoride (PMSF; ThermoFisher, cat# 36 978), 40 mM imidazole], and the protein was eluted with Ni-B buffer (40 mM Tris–HCl, pH 8, 5% glycerol, 50 mM KCl, 10 mM β-ME, 500 mM imidazole). The eluted protein was loaded onto the HiTrap Heparin HP column (Cytiva, cat# 17040701) and eluted with a linear gradient of KCl (0–1 M) in Hep buffer [20 mM Tris–HCl, pH 8, 5% glycerol, 1 mM tris(2-carboxyethyl)phosphine (TCEP)].

*Hha*: The cell pellet was resuspended in Lysis buffer B [100 mM HEPES-KOH, pH 7.5, 300 mM KCl, 5% glycerol, 1× cOmplete protease inhibitor cocktail (EDTA-free; Roche Diagnostics, cat# 11836170001), 10 mM imidazole, 5 mM β-ME), and lysed by sonication. The cleared lysate was applied to Ni^2+^-NTA resin (Cytiva, cat# 17531801), washed with Ni-C buffer (20 mM HEPES-KOH, pH 7.5, 300 mM KCl, 5% glycerol, 5 mM β-ME, and 0.1 mM PMSF) supplemented with 30 mM imidazole, and eluted with Ni-buffer D (20 mM HEPES-KOH, pH 7.5, 50 mM KCl, 5% glycerol, 5 mM β-ME, 0.1 mM PMSF, 300 mM imidazole). The eluted protein was buffer exchanged to SEC buffer (20 mM HEPES-KOH, pH 7.5, 300 mM KCl, 5% glycerol, 5 mM β-ME) with a HiPrep 26/10 desalting column (Cytiva, cat# 17 508 701).

*Sfx*: The cell pellet was resuspended in Lysis buffer A and opened by sonication. The cell lysate was cleared by centrifugation (20 000  ×  *g*) for 30 min at 4°C. The supernatant was passed through a 0.45 μm filter and applied to High Affinity Ni-charged Resin (Genscript). The resin was washed with Ni-A buffer, and protein was eluted with Ni-C buffer (40 mM Tris–HCl, pH 8, 5% glycerol, 0.5 mM KCl, 10 mM β-ME, and 500 mM imidazole). The eluted protein was loaded onto a desalting column (Cytiva) in desalting buffer (40 mM Tris–HCl, pH 8, 5% glycerol, 1 mM TCEP, and 0.4 M KCl) to remove imidazole.

His-SUMO-tagged H-NS, Hha, and Sfx proteins were treated with ULP1 protease (purified in-house) at 4°C overnight to remove the tag. The sample was passed through Ni-charged resin again to remove the ULP1 protease and the tagged proteins. The untagged protein was loaded onto a Superdex 75 10/300 GL column (Cytiva, cat# 17517401). Protein purity was assessed by sodium dodecyl sulfate–polyacrylamide gel electrophoresis (SDS–PAGE) and Coomassie staining. Protein concentrations were determined by measuring the absorbance at 280 nm on a Nanodrop ND-1000 spectrometer. Purified proteins were aliquoted and stored at −80°C.

### Protein labeling

A single cysteine residue was added to the C-terminus of H-NS and Sfx (Supplementary Table S1). The proteins were first purified as above without cleaving the His-SUMO tag, followed by on-bead labeling. The His-tagged proteins were first reduced by incubating with Ni-charged MagBeads (GenScript, cat# L00295) in desalting buffer for 3 h at 4°C on a rotating wheel. Then, the magnetic beads were washed with phosphate buffered saline (PBS) buffer (137 mM NaCl, 2.7 mM KCl, 8 mM Na_2_HPO_4_, and 2 mM KH_2_PO_4_, pH 7.4; degassed for half an hour before use) and resuspended in PBS. Fluorescent dyes were diluted 75 times into the resuspended beads. Alexa Fluor 546 C5 Maleimide (AF546; Thermo Fisher, cat# A10258) and Cyanine 5 maleimide (Cy5; AAT Bioquest, cat# 152) were used for Sfx and H-NS derivatives, respectively. After overnight incubation at 4°C, the beads were washed with desalting buffer and then treated with ULP1 for 4 h at 4°C to release the labeled proteins from magnetic beads. Protein purity was assessed by SDS–PAGE, and the protein concentration was determined by the Bradford protein assay. The proteins were aliquoted, flash frozen in liquid nitrogen, and stored at −80°C.

### Reverse transcription quantitative PCR

*Escherichia coli* BW25113 carrying R6K or R6KΔ*sfx* were cultured in LB with carbenicillin. The cells were grown at 37°C to an OD_600_ of ∼0.7 and treated with bicyclomycin (BCM; 50 μg/ml) for 15 min, where indicated. The cells were mixed with 0.2 volume of ice-cold growth stop solution (5% phenol and 95% ethanol) to inactivate cellular RNase before being collected by centrifugation at 4000  ×  *g* for 10 min. The messenger RNA (mRNA) was isolated using the Monarch Total RNA Miniprep Kit (NEB, cat# T2010S). RNA samples were treated with TURBO DNase (Thermo Fisher, cat# AM2239) before RT-qPCR. A total of 100 ng RNA samples was used with iTaq Universal SYBR Green One-Step Kit (Bio-Rad, cat#1725150) and analyzed on a CFX96 system (Bio-Rad). Samples without reverse transcriptase were used as a negative control to ensure the absence of DNA contamination. The quantification cycle (*C*_q_) values were calculated using CFX Manager v3.0 in regression mode. The gene expression level was analyzed by the threshold cycle (2^−ΔΔCT^) method [31]; the *ihfB* gene was used as a reference, expression of which is not affected by BCM [32].

### Next-generation sequencing sample preparation

*RNA-seq samples: Escherichia coli* BW25113 carrying R6K or R6KΔ*sfx* were grown in LB medium supplemented with carbenicillin at 37°C and 250 rpm. Cells were collected by centrifugation when the OD_600_ reached 0.6. Total RNA was first extracted with RNAzol RT (Millipore Sigma, cat# R4533), and the top water phase was fed into Monarch Total RNA Miniprep Kit (NEB). The extracted RNA was further treated with DNase I (NEB, cat# M0303S). The ribosomal RNA (rRNA) was removed by using NEBNext rRNA Depletion Kit (Bacteria) (NEB, cat# E7850S) following the manufacturer’s protocol. The RNA samples were submitted for stranded RNA sequencing.

*Sfx and H-NS ChIP-seq samples*: Chromatin immunoprecipitation followed by sequencing (ChIP-seq) was used to identify Sfx and H-NS binding sites. The procedure was adapted from a previous publication [33]. FLAG tag was added to the C-terminus of H-NS and Sfx. The overnight cultures (∼16 h) of cells containing either H-NS-3xFLAG encoded on the chromosome or Sfx-FLAG expressed from a low-copy plasmid under its native promoter (p451) (Supplementary Table S1) were mixed with formaldehyde/sodium phosphate (pH 7.4) buffer to yield a final concentration of 10 mM NaPO_4_ and 1% (v/v) formaldehyde. The 20 ml cells were crosslinked for 5 min at 37°C, 250 rpm. The crosslinking was stopped by adding 0.33 M glycine, followed by a 30-min incubation in an ice-water bath. The crosslinked cells were collected by centrifugation, washed three times with PBS, and resuspended in 1× IP buffer [200 mM Tris–HCl, pH 8, 600 mM NaCl, 4% Triton X-100, and 2× ProBlock Gold 2D Protease Inhibitor Cocktail (Goldbio)]. The resuspended cells were subjected to controlled sonication to yield DNA fragments ranging from 100 to 500 bp. The cells were kept in an ice-water bath throughout sonication.

After sonication, 0.1 volume of cell lysate, which is used as the input sample, was kept at 4°C until the reversal of crosslinking. The remaining cell lysate (ChIP sample) was mixed with 30 μl of MonoRab anti-DYKDDDDK magnetic beads (GenScript, cat# L00835-1). After an overnight incubation at 4°C, the magnetic beads were subjected to sequential washes, with 1 ml used per wash: (i) 1× Wash buffer A (100 mM Tris–HCl, pH 8, 250 mM LiCl, 2% Triton X-100, 1 mM EDTA); (ii) 1× Wash buffer B (100 mM Tris–HCl, pH 8, 500 mM NaCl, 1% Triton X-100, 0.1% sodium deoxycholate, 1 mM EDTA); (iii) 1× Wash buffer C (10 mM Tris–HCl, pH 8, 500 mM NaCl, 1% Triton X-100, and 1 mM EDTA); (iv) 1× TE (10 mM Tris–HCl, pH 8, 1 mM EDTA). The antigens were eluted in 1 ml of elution buffer [50 mM Tris–HCl, pH 8; 10 mM EDTA; 1% SDS, 5 U/ml proteinase K (NEB, cat# P8107S)] during 1-h incubation at 65°C, with vigorous vortexing every 10 min. The input samples were diluted 10 times with elution buffer and subjected to the same treatment as the ChIP samples from now on. After removing the magnetic beads from ChIP samples, both ChIP and input samples were incubated at 65°C overnight to reverse the crosslinking. To digest the RNA, 100 μg/ml of RNase A (Thermo Fisher, cat# EN0531) was added for a 2-h incubation at 37°C, followed by 2-h incubation at 50°C with 5 U/ml of proteinase K (NEB). Finally, DNA was recovered using QIAquick PCR Purification Kit (QIAGEN, cat# 28104). Recovered DNA samples were submitted for sequencing.

*RNAP ChIP-seq samples: Escherichia coli* MG1655 cells harboring R6KΔ*sfx* were grown in 60 ml LB at 37°C, 250 rpm, and were treated with 150 μg/ml rifampicin for 10 min at OD_600_ ∼0.4. The treated cells were crosslinked and washed as earlier. The protocol for immunoprecipitation is largely the same as Sfx ChIP-seq sample preparation except for the incubation with antibodies. The samples were first incubated with 30 μl of Protein A/G MagBeads (GenScript, cat# L00277) for 3 h at 4°C, and the protein A/G MagBeads were then removed. Five microliters of anti-*E. coli* RNA polymerase β subunit antibody (abcam, cat# ab191598) was added to the samples for an overnight incubation at 4°C. The next day, 30 μl of Protein A/G MagBeads were added. After 3 h of incubation, the magnetic beads were washed, and the DNA was extracted as earlier.

*RNA ChIP-seq samples*: The protocol is largely the same as the Sfx ChIP-seq sample preparation, with the following changes. After reverse crosslinking, the samples were treated with Turbo DNase (Thermo Fisher) instead of RNase A, and the RNA was purified by Monarch Total RNA Miniprep Kit (NEB) following the manufacturer’s protocol. The resulting RNA was submitted for stranded RNA sequencing.

All samples were submitted to IDI-GEMS laboratory, The Ohio State University, for next-generation sequencing (NGS). To generate libraries for DNA samples, the Illumina DNA Prep kit (Illumina, cat# 20060060) was used following the procedure for selecting <500 bp fragments. Samples were barcoded using IDT for Illumina UD Indexes. Stranded Total RNA Library Prep (Illumina, cat#20040525) was used for RNA samples following Illumina’s protocol. Library concentration was measured using a Qubit Flex fluorometer (Thermo Fisher), and the library fragment size was analyzed using a TapeStation 4150 (Agilent).

### NGS data analysis

*RNA-seq*: Adapter and low-quality reads were trimmed using TrimGalore v0.6.7 [34] in paired-end mode, and one base was trimmed from the 5′ end of all reads (Parameters: –paired –trim-n -clip_R1 1 –clip_R2 1). Reads were then aligned using bowtie2 v2.4.4 [35] with very sensitive, end-to-end alignment, and dovetailed alignments allowed (Parameters: –very-sensitive –no-mixed –no-discordant –dovetail -X 1000). The reads were mapped to *E. coli* K-12 substr. BW25113 (ASM75055v1) and plasmid R6KΔ*sfx* (pIA1548; Dataset 2). The R6K sequence used in this study is the same as NCBI ID LT827129.1, except that we shifted the coordinates to group the high GC-region for visualization (Fig. 1A, Dataset 2). The alignment files were converted to BAM format and indexed by SAMtools v1.23 [36]. The alignment files were subjected to quantification using featureCounts v2.0.3 [37] with the –countReadPairs option. Finally, the differential expression was analyzed using DEseq2 v3.22 [38]. The IGV tracks were generated using deepTools v3.5.6 [39].

*ChIP-seq*: Adapter and low-quality reads were trimmed using TrimGalore v0.6.7 [34] in paired-end mode (Parameters: –paired –trim-n), and “–2color 30″ was added to the parameters if poly-G artifacts were present in the reads. HupB ChIP-seq reads were trimmed in single-end mode by omitting the –paired flag. For RNA ChIP-seq reads, parameters “–trim-n -q 28 –clip_R1 1 –clip_R2 1 –paired” were used. Trimmed reads were aligned to *E. coli* BW25113 (ASM75055v1) and plasmid R6KΔ*sfx* (pIA1548) using bowtie2 v2.4.4 [35] with very sensitive, end-to-end alignment, and dovetailed alignments allowed (Parameters: –very-sensitive –no-mixed –no-discordant –dovetail -X 1000). HupB reads were aligned with parameters “–very-sensitive -q -U.” The alignment files were converted to BAM format and indexed by SAMtools v1.23 [36]. Peak calling was done by MACS3 v3.0.3 [40] using the subcommand of callpeak (Parameters: -f BAMPE -B –trackline -g 4 675 061 –keep-dup auto -q 0.01). For RNA ChIP-seq, parameters “-f BAMPE -B –trackline -g 4 675 061 –keep-dup all –call-summits –nomodel –extsize 140” were used. HupB binding peaks were called without a control sample (Parameters: -f BAM -B –trackline -g 4 675 061 –keep-dup auto -q 0.01 –nomodel –broad). The MACS plots in all figures are the binding region consensus of two biological repeats. Searching for binding motifs of H-NS and Sfx was done by HOMER v5.1 [41] using peaks on the chromosome. The tracks displayed in IGV v2.19.7 [42] were calculated by MACS3 bdgcmp and converted to bigWig files by bedGraphToBigWig v2.10 [43]. The −10 and −35 elements of promoters were predicted by BPROM [44], and the transcription start site was predicted by BDGP [45]. The Spearman and Pearson correlations were calculated using multiBigwigSummary and plotCorrelation from deepTools v3.5.6 [39]. Searching the high-affinity binding sites (named Seeds) of Sfx on R6K was done by Fuzzy Search DNA [46] using default settings with the pattern AATAATAAATATAT identified in this study as a query.

### ChIP-qPCR

The samples were prepared as Sfx and H-NS ChIP-seq sample preparation. The resulting DNA was submitted for qPCR instead of NGS. The qPCR was performed on a CFX96 system (Bio-Rad) with iTaq Universal SYBR Green Supermix (Bio-Rad, cat# 1725120). Primers for qPCR are listed in Supplementary Table S1. One nanogram of DNA was loaded into each well. The Cq values were calculated by CFX Manager v3.0 using regression mode. The fold enrichment (FE) of the target fragment was analyzed by the threshold cycle (2^−ΔΔCT^) method [31].

### Electrophoretic mobility shift assay

Linear DNA templates were generated by PCR amplification (Supplementary Table S1) and purified using QIAquick PCR Purification Kit (QIAGEN). SsrA RNA was generated by *in vitro* transcription with T7 RNA polymerase P266L mutant [47]. The reaction in 40 mM Tris–HCl, pH 7.9, 10 mM NaCl, 6 mM MgCl_2_, 10 mM DTT, 2 mM spermidine, 0.05% Tween 20, and 0.5 mM of each nucleoside triphosphate (ATP, CTP, GTP, and UTP) was incubated at 30°C for 2.5 h. After treatment with TURBO DNase (Thermo Fisher), RNA was purified by Monarch Spin RNA Cleanup Kit (NEB, cat# T2030S). Radioactive labeling of DNA and RNA templates was done using T4 PNK (NEB) and ATP [γ-^32^P] (Revvity, cat# BLU002Z).

Indicated protein and template were incubated in electrophoretic mobility shift assay (EMSA) buffer (25 mM Tris–HCl, pH 7.5, 100 mM KCl, 5 mM MgCl_2_, and 1 mM TCEP) at room temperature (∼23°C) for 15 min. Then the reactions were mixed with 20× loading buffer (30% glycerol, 0.2% Orange G) and separated on 3% polyacrylamide or 1% agarose gels in 0.5× TBE (Research Products International, cat# T22020) at 4°C. The DNA and RNA species were visualized by Amersham Typhoon 5 and quantified by Fiji v2.17.0 [48].

### Pelleting assay

Reactions containing different protein, plasmid, and RNA combinations were assembled in 20 μl EMSA buffer. After a 15-min incubation at room temperature, the reactions were centrifuged at 21 000  ×  *g* for 25 min at 20°C. The supernatant was removed, and the pellet was soaked in 15 μl of HSB buffer (20 mM Tris–HCl, pH 7.5, 5 mM DTT, 500 mM NaCl) and 5 μl of 4× LDS sample buffer (GenScript, cat# M00676) for 30 min at room temperature before resuspending. The samples were analyzed on SurePAGE 4%–12% gels (GenScript, cat# M00654) and stained with GelCode Blue Safe Protein Stain (Thermo Fisher, cat# 24594). The gels were visualized by Amersham Typhoon 5 and quantified by Fiji v2.17.0 [48].

### Plasmid copy number determination

Plasmid copy number was determined by a qPCR-based method modified from a previously published protocol [49]. The *rpoC, bla*, and *cat* genes were selected as target genes for the chromosome, R6K, and p451, respectively. The amplification efficiencies of primers of *rpoC, bla*, and *cat* are determined to be ∼1 (Supplementary Table S1). About 10^9^ cells were collected by centrifugation and resuspended in 100 μl of 20 mM Tris–HCl, pH 8, 20 mM EDTA, 0.2% SDS. After incubating for 5 min at 95°C to open the cells, RNase A (Thermo Fisher) was added to a final concentration of 0.2 μg/μl. The reaction was incubated for 1 h at 37°C, followed by incubation with 5 U/ml Protease K (NEB) for 1 h at 50°C. Finally, the sample was incubated for 10 min at 95°C and centrifuged at 20°C, 21 000  ×  *g* for 10 min to pellet insoluble fibers. The cleared supernatant was diluted 500 times with DNase-free H_2_O, and 1 μl of the diluted sample was used for qPCR. The iTaq Universal SYBR Green Supermix (Bio-Rad) was used for qPCR analysis on the CFX96 system. The plasmid copy number is expressed as the ratio of plasmid gene copy to chromosomal gene copy, which was calculated using the threshold cycle (2^−ΔΔCT^) method [31].

### Conjugation assays

*Escherichia coli* BW25113 strains harboring R6K derivatives were used as donors, and *E. coli* BW25113 *lysA*::Kn^R^ strain was used as the recipient. For filter-paper mating, donors and recipients were grown overnight in LB supplemented with selective antibiotics at 37°C, 250 rpm. Overnight cells were collected by centrifugation, and the pellet was washed once with sterile PBS. Following the wash, 10^9^ donor cells and 10^9^ recipient cells were mixed and dropped onto an MCE membrane filter (0.22 µm pore size; Millipore Sigma, cat# GSWP01300) overlaid on LB agar and incubated at 30°C for 2 h. The filter paper was then vortexed in 1 ml of sterile PBS. Serial dilutions of each sample were spotted on M9 (ATCC Medium 2511) + carbenicillin, LB + kanamycin, and LB + kanamycin + carbenicillin to select for donor, recipient, and transconjugant populations, respectively.

Filter-paper mating and liquid mating were used for testing the effects of novobiocin on conjugation. *Escherichia coli* BW25113 *argE*::tet^R^ carrying R6K75 (a transposon-deletion R6K; Supplementary Table S1, Dataset 2) or R6K75Δ*sfx* were used as donors, and *E. coli* BW25113 *lysA*::Kn^R^ was the recipient. The filter-paper mating assay follows the above procedure with modifications: overnight cells were treated with novobiocin (250 μg/ml) for 1 h before 1 h mating. For the liquid mating assay, the overnight cells were reinoculated into fresh medium. At an OD_600_ of 0.4, cultures were exposed to novobiocin (250 μg/ml) for 1 h, followed by washing once with sterile PBS. Five hundred microliters of culture mixtures in PBS containing 10^9^ donor cells and 10^9^ recipient cells were incubated at 37°C for 30 min, then vortexed to stop conjugation. Serial dilutions of each sample were then spotted on LB + tetracycline + carbenicillin, LB + kanamycin, and LB + kanamycin + carbenicillin to select for donor, recipient, and transconjugant populations, respectively. Plates were incubated overnight at 37°C.

### Western blot

*Escherichia coli* MG1655 carrying FLAG-tagged *sfx* or *hns* was cultured overnight in LB + carbenicillin, and the cells were collected by centrifugation. The cells were opened by sonication in 25 mM Tris–HCl, pH 7.4, 7 M urea, and followed by resolving in 4%–12% SurePAGE gel (GenScript). Then, the proteins were transferred to a nitrocellulose membrane (Bio-Rad, cat# 1620112) in a Trans-Blot SD Semi-Dry Transfer Cell (Bio-Rad) with 15 V for 15 min in transfer buffer (25 mM Tris–HCl, pH 8.3, 192 mM glycine, and 20% methanol). After the transfer step, the membrane was blocked in blocking buffer [25 mM Tris–HCl, pH 7.4, 150 mM NaCl, 0.1% Tween-20, and 5% (w/v) blotting grade blocker non-fat dry milk (Bio-Rad, cat# 706404XTU)] for 1 h at room temperature. The membrane was washed twice with TBST (25 mM Tris–HCl, pH 7.4, 150 mM NaCl, 0.1% Tween-20), followed by incubation with anti-FLAG polyclonal antibodies (1:10 000) (Millipore Sigma, cat# F7425) in blocking buffer overnight at 4°C. The next day, the membrane was washed five times with TBST and incubated with secondary antibody [1:10 000; Goat Anti-Rabbit IgG (H + L)-HRP Conjugate (Bio-Rad, cat# 1706515)] in blocking buffer for 1 h at room temperature. The membrane was then washed five times with TBST. Finally, the membrane was incubated with Clarity Max Western ECL Substrate (Bio-Rad) for 5 min at room temperature and imaged with ChemiDoc XRS + System (Bio-Rad). Quantification was done with Image Lab v6.1 (Bio-Rad).

### Yellow fluorescent protein activity assay

*Escherichia coli* BW25113 was used for the YFP activity assay. To test the effects of Sfx on the *actX* promoter (P*actX*), a YFP reporter was constructed by putting the *yfp* gene directly downstream of P*actX* (pIA1723; Supplementary Table S1). In order to test the *in trans* effects of Sfx, the P*actX*-*yfp* reporter was transformed into *E. coli* together with R6K or R6KΔ*sfx*. To establish the background, a control plasmid pBAD33 (p28; Supplementary Table S1), conferring the same chloramphenicol resistance as pIA1723, was transformed into *E. coli* together with R6K or R6K*Δsfx*. Five biological replicates of each strain were grown overnight in LB supplemented with carbenicillin and chloramphenicol at 37°C, 250 rpm. The next morning, 1 μl of each replicate was used to inoculate 150 μl fresh media on a sterile, black-bottom 96-well plate (Greiner Bio-One, cat# 655077). Cells were allowed to grow for 24 h at 37°C with double-orbital shaking at 250 rpm in the VANTAstar plate reader (BMG). YFP fluorescence signal reads were taken every 15 min, and the final read was used for analysis. OD_600_ was used to normalize the fluorescence results.

To test the effects of Sfx *in cis*, P*actX*-*yfp* reporter was inserted into R6K or R6KΔsfx (pIA1767/1768; Supplementary Table S1). *Escherichia coli* carrying R6K and *E. coli* carrying R6KΔsfx were used as background control. Cells were grown in LB supplied with carbenicillin. The testing procedure is the same as earlier.

### Atomic force microscopy

Shortly before deposition of the sample, a 5 μl droplet of 0.01 ug/ml of poly-L-ornithine (1 kDa MW, Millipore Sigma) was deposited onto freshly cleaved mica and incubated for 2 min. The poly-L-ornithine-coated mica was rinsed drop-wise with 400 μl of Nanopure (18 Mohm) water and dried gently with a stream of air from a portable compressor. Then, 5 μl of the sample solution containing 0.4 nM DNA plasmids was deposited on the poly-L-ornithine-coated mica and incubated for 2 min. This droplet was rinsed with 400 μl of Nanopure water and dried gently with compressed air. Images were acquired with a NanoScope MultiMode VIII AFM microscope (Bruker Nano Surfaces) operating in Tapping Mode using MicroMasch NSC:18 cantilevers with 5 nm nominal tip radius. Areas of 2 × 2 μm were scanned at a rate of ∼0.25 Hz with a resolution of 1024 × 1024 pixels. Images were filtered to remove scan line offsets and tilt/bow.

### Microscopy

Microscopy‐based experiments were carried out in a Metamorph-driven Olympus BX-61. Glass slides (1 mm thickness; Thermo Fisher, cat# 125444) and coverslips (0.17 mm thickness; VWR, cat# 48366-249) were washed with milliQ water (18 Mohm) and 70% ethanol, and dried in a 42°C oven overnight. The dried glass slides and coverslips were coated with 1% (w/v) Pluronic F-127 (PF127; Millipore Sigma, cat# P2443). PF127 was prepared freshly in milliQ water. The treated slides and coverslips were rinsed gently with milliQ water and allowed to air-dry for 30 min. The proteins and R6K/RNA were mixed in a 10 μl reaction in EMSA buffer supplemented with polyethylene glycol 8000 (PEG8000). After 10 min at room temperature, the whole 10 μl was dropped onto the PF127-coated glass slide and covered with a PF127-coated coverslip. The droplets were visualized using a 40× objective in either bright field or fluorescence modes using an appropriate fluorescence channel.

## Results

### Sfx promotes early termination in the R6K vir operon but has little effect on chromosomal genes

Conjugation is a fundamentally important process that drives horizontal gene transfer. Studies of IncX plasmids focused on the regulation of the major promoter of the conjugal transfer operon [22, 50]. Although R6K has been used as a conjugation model for decades [51], the promoters of its *vir* operon are still unknown. To understand R6K conjugation regulation, we first used RNAP ChIP-seq to identify potential promoters in R6K (Supplementary Fig. S1) upon treatment with rifampicin, an RNAP inhibitor that traps RNAP at promoters [52]. We observed RNAP peaks upstream from *pir, virBR*, antibiotic resistance genes, and the *actX* gene, which could mark potential promoters. Based on studies of other IncX plasmids, P*actX* is expected to function as the main *vir* operon promoter [22, 50], and its identification is also supported by our RNA-seq data (Fig. 1C, Dataset 1B–D) and *in silico* promoter prediction.

Recent reports used GFP and LacZ reporters to demonstrate that IncX3 conjugation is controlled during initiation by Sfx homologs pHNS/HppX3, which act as repressors of the P*actX* promoter region [22, 50, 53]. We hypothesized that Sfx could also (or instead) control the *vir* operon expression during elongation; long xenogeneic operons such as the *vir* operon (∼13 kb) are frequently silenced by Rho, which can act together with H-NS to induce premature RNA release during transcription of the chromosomal targets [11, 54–56]. To determine if Sfx inhibits RNAP recruitment to P*actX*, we constructed reporters in which the P*actX* promoter (−218 to +114) was fused to the *yfp* gene and tested in the presence (or absence) of the R6K-encoded Sfx (Fig. 1D). We compared the effects of Sfx expressed from R6K either *in trans*, with the reporter on a P15A plasmid (Fig. 1D top), or *in cis* on R6K (Fig. 1D bottom). In both cases, we observed robust YFP activity, which was not suppressed by Sfx, suggesting that Sfx controls the *vir* operon during elongation.

To evaluate this possibility, we tested the effects of Sfx and Rho on *vir* operon expression using RT-qPCR. Deletion of the *sfx* gene led to the upregulation of the four selected *vir* genes, whereas the chromosomal *rho* gene was not affected (Fig. 1E). Inhibition of Rho activity by bicyclomycin (BCM) increased the *rho* RNA levels 30 times, consistent with the autoregulation of the *rho* gene [57], as well as upregulation of the *vir* operon (Fig. 1E). Deleting *sfx* decreased the effects of BCM treatment (Fig. 1E). We conclude that Rho and Sfx synergistically repress the R6K conjugation operon. Our results, along with the presence of genes that encode RfaH homologs on many conjugative plasmids, including R6K, suggest that transcription elongation control is a common regulatory strategy among these plasmids.

Some plasmid-encoded H-NS homologs have been reported to act as back-up copies of H-NS; e.g. Sfh, encoded by the *Shigella flexneri* IncHI1 plasmid, shares the chromosomal binding sites and effects on gene expression with H-NS [26, 27]. To identify R6K and host genes controlled by Sfx, we performed RNA sequencing. Our results show that only a handful of genes are significantly affected by the *sfx* deletion in the log phase (Fig. 1F, Dataset 1B). Among these genes, only two transfer RNA genes located in the same *E. coli* operon were modestly downregulated, whereas all other genes were upregulated, consistent with the silencing role of Sfx. On R6K, most of these upregulated genes encode conjugation functions (Fig. 1A and F). On the chromosome, three genes (*yhjX, btsT*, and *tnaC)* were significantly upregulated (Fig. 1F), all of which are implicated in pyruvate metabolism. The *tnaAB* operon leader peptide *tnaC* is significantly upregulated, but changes of *tnaA and tnaB* are not significant enough (FDR = 0.03), although they stand out from the chromosomal genes (Fig. 1F). Studies of ColE1 plasmid maintenance suggested that indole produced by the *tnaCAB* operon can inhibit cell division and plasmid replication to allow for multimer resolution [58]. We speculate that the upregulation of *tnaCAB* increases pyruvate and indole production [59, 60], which in turn could activate the BtsS/BtsR system, inducing *btsT* and *yhjX* to manage pyruvate homeostasis [61–63]. Thus, *btsT* and *yhjX* changes might be caused by the *tnaCAB* operon.

Our results demonstrate that Sfx selectively silences the plasmid genes, whereas the published data show that H-NS controls many chromosomal genes [64], but does not inhibit R6K conjugation [24]. To understand how these homologous silencers are directed to their respective regulons, we next determined the binding sites of Sfx and H-NS.

### Sfx weakly binds to many H-NS loci on the chromosome

**Figure 2.**
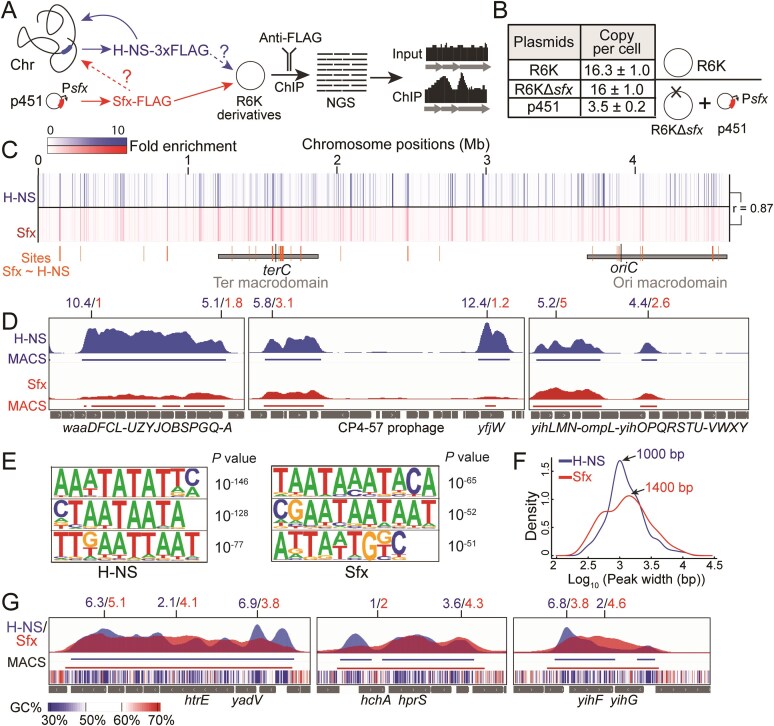
Sfx and H-NS share binding sites on the chromosome. (**A**) Schematic of the H-NS and Sfx ChIP-seq assay. Chr, chromosome. NGS, next-generation sequencing. (**B**) Plasmid copy numbers are shown as mean ± SD (*n* = 5). (**C**) Heatmap tracks of chromosome binding sites. Spearman correlation (*r*) analysis was performed on reads binned at 300 bp. Below the tracks, the two vertical black bars represent the replication terminus (*terC*) and origin (*oriC*). Orange bars are sites of similar Sfx and H-NS enrichment (Sfx > 70% of H-NS). Two horizontal gray bars are the chromosomal macrodomains. (**D**) Examples of H-NS and Sfx binding sites. The peaks in tracks represent FE, which ranged from 0 to 13. The tracks in all figures are the average of two biological repeats unless stated otherwise. For selected sites, H-NS and Sfx peak heights in this and subsequent figures are indicated in blue and red, respectively. In all panels, the MACS plot below the ChIP-seq profiles shows statistically significant regions bound by H-NS (blue) or Sfx (red). (**E**) The binding motifs of Sfx and H-NS were predicted from the peaks on the chromosome by HOMER. The top three motifs with the lowest *P* values are shown. (**F**) Density plot of the log_10_(width) of H-NS and Sfx peaks on the chromosome. The peak widths of the highest density are indicated. (**G**) Examples showing Sfx peaks extending through the valleys of H-NS peaks. The GC content, calculated in a 25-bp sliding window, is shown as a heatmap. The peaks in tracks represent FE, which ranged from 0 to 8.

We used ChIP-seq to analyze the genome-wide binding profile of H-NS and Sfx (Fig. 2A). H-NS carries the C-terminal 3×FLAG tag, which does not substantially perturb its function [65, 66]. Sfx is expressed from its native promoter on a pSC101-derived plasmid (referred to as p451; Supplementary Table S1) and carries a single FLAG tag. Unlike the triple-tagged Sfx, the plasmid-borne Sfx-1×FLAG variant is fully active in silencing R6K conjugation (Supplementary Fig. S2A and B). We have determined the copy numbers of plasmids in the stationary phase, the conditions used for conjugation and ChIP-seq assay (Fig. 2B): p451 is present at 3–4 copies/cell, and R6K at 16 copies/cell; the latter number is within the published range [67]. Our finding that Sfx can silence conjugation when expressed from the ∼4-fold less abundant locus (Fig. 2B and Supplementary Fig. S2B) suggests that Sfx expression may be autoregulated, as reported for H-NS [68].

On the chromosome, peaks of Sfx and H-NS largely overlap (with Spearman correlation of 0.87), though H-NS displays higher enrichment values at almost all loci (Fig. 2C). For example, while H-NS has ∼10-FE on the LPS core biosynthesis *waa* operon and *yfjW* gene of the CP4-57 prophage, Sfx has <1.5-FE (Fig. 2D, Dataset 1E–H). The sites that show similar Sfx and H-NS peaks are mainly located in the replication terminus and origin macrodomains (Fig. 2C); both H-NS and Sfx have ∼5-FE on the *yih* operon (Fig. 2D). We note that these data cannot be used to compare true occupancies of the two proteins due to differences in their cellular concentrations, immunoprecipitation efficiency, and formaldehyde crosslinking between the proteins and across different DNA regions, a common limitation in ChIP studies. In addition, some of the observed shared sites may represent mixed filaments, which H-NS has been shown to form with its other homologs [21]. Thus, here we focus on comparing the profile differences between H-NS and Sfx.

To estimate the relative protein concentrations of H-NS and Sfx under the conditions used for the ChIP-seq analysis, we used western blotting with anti-FLAG antibodies. Our results suggest that the molar ratio of Sfx/H-NS is at least 3.5 when Sfx-FLAG is expressed from p451, and at least 3.7 when Sfx-FLAG is expressed from R6K (Supplementary Fig. S2C). The higher abundance of Sfx is expected to be offset by the reduced affinity for the anti-FLAG antibodies; the manufacturer’s technical notes state that the 3×FLAG increases the detection limit in western blots by at least 10-fold compared to 1×FLAG. If this preference persists during immunoprecipitation, the observed H-NS/Sfx signal ratio might be overestimated by approximately three-fold.

Selective protein targeting may be achieved through recognition of distinct DNA elements. Numerous studies of H-NS interactions with the DNA support the notion that H-NS prefers AT-rich sequences with loose specificity [69, 70]; following its loading at a high-affinity binding site, H-NS cooperatively spreads to adjacent regions [10, 71, 72]. The majority of Sfx and H-NS peaks on the chromosome appear to coincide (Fig. 2C), suggesting that Sfx may recognize similar DNA motifs. We analyzed the Sfx and H-NS chromosomal peaks to compare their binding motifs; the top three motifs shown in Fig. 2E reveal that both proteins prefer AT-rich DNA, and their binding motifs can be part of a shared pattern AATAATAAATATAT (Fig. 2E). Interestingly, Sfx appears to tolerate more GC residues, and this property may partially explain its tendency to spread along the DNA in a more uniform pattern (see below).

Sfx has a higher chance of forming broader peaks than H-NS. While H-NS displays a maximum probability at 1000 bp peak width, Sfx peaks at 1400 bp and exhibits a higher probability of reaching widths beyond 3000 bp (Fig. 2F). Sfx also forms narrower peaks, which could be explained by the low FE. The peak boundary is determined by MACS3 [40] based on *P* values, and a low FE can result in a non-significant *P* value, pushing the boundary toward the peak center and producing narrower peaks. The wider peaks of Sfx are the result of shallow peak valleys. For example, chromosomal regions shown in Fig. 2G contain multiple H-NS peaks separated by deep valleys, whereas Sfx peaks are connected by shallow valleys and thus appear broadened. The same pattern is observed genome-wide.

### Sfx and H-NS have similar preferences for DNA topology

**Figure 3.**
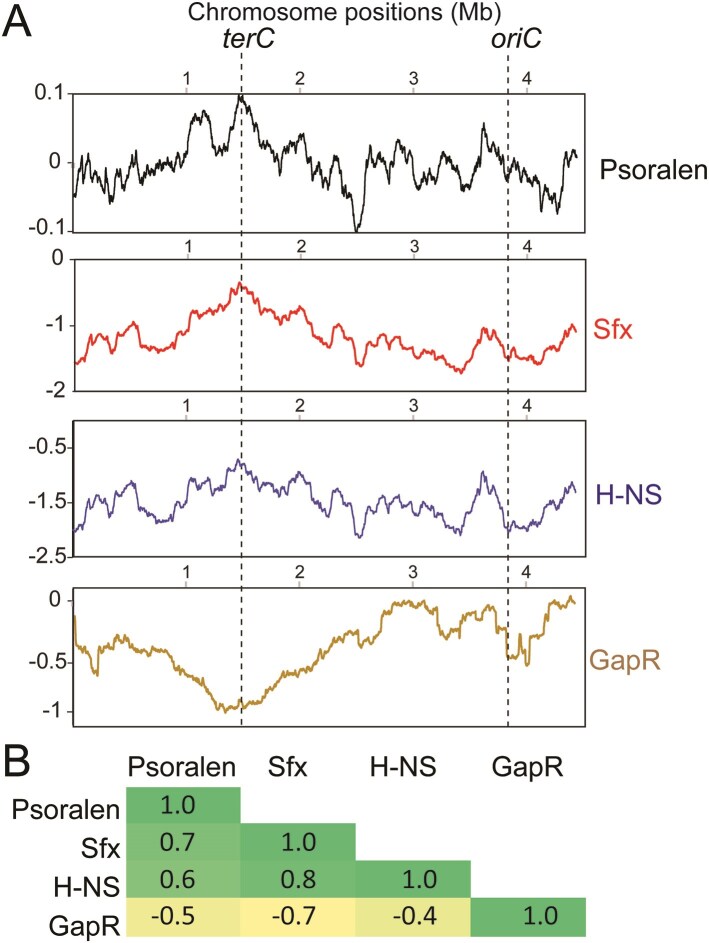
Sfx prefers negatively supercoiled DNA. (**A**) Correlation between DNA topology and Sfx/H-NS binding sites. The *Y*-axis values represent the moving average of 200-kbp windows. Psoralen plot is the log-transformed ratio of psoralen crosslinked to non-crosslinked fraction. Sfx/H-NS/GapR plots are the mean values of log_2_FE of two biological repeats. FE = ChIP/Input. Positions of the chromosome replication terminus (*terC*) and origin (*oriC*) are indicated. (**B**) Pearson correlation between the calculated moving average in panel (A).

Since H-NS is known to preferentially bind to negatively supercoiled DNA [73–77], similar ChIP-seq peak locations (Fig. 2C) led us to assume that Sfx shares this preference. To further evaluate this premise, we compared H-NS and Sfx binding profiles to those of psoralen [78] and GapR [79] (Fig. 3A). Psoralen and GapR selectively bind to negatively- and positively-supercoiled DNA, respectively [79, 80]. Our analysis revealed that H-NS and Sfx profiles were similar to that of psoralen, yet quite distinct from the GapR profile (Fig. 3A), supporting the conclusion that both H-NS and Sfx prefer negatively supercoiled DNA. Consistently, the H-NS and Sfx profiles have a positive Pearson correlation with psoralen, but a negative correlation with GapR (Fig. 3B).

To determine whether Sfx activity in the cell depends on DNA topology, we used novobiocin, which inhibits DNA gyrase and negative supercoiling [81]. We carried out conjugation assays with donors carrying the WT and Δ*sfx* R6K following a 1-h treatment with novobiocin. Our results show that the inhibition of DNA gyrase dramatically (>100-fold) increases the conjugation efficiency of WT R6K to match the level of R6KΔ*sfx* but has no effect on that of R6KΔ*sfx* (Supplementary Fig. S3). These results indicate that, somewhat surprisingly, the loss of negative supercoiling does not compromise the DNA processing and transfer functions but abolishes Sfx-mediated silencing (and likely targeted binding).

### Similarly to H-NS, Sfx bridges DNA *in vitro* and likely *in vivo*

Our results demonstrate that Sfx silences the *vir* operon during RNA chain elongation (Fig. 1E). The published data show that the H-NS ability to induce premature termination depends on the formation of bridged H-NS/DNA filaments [21, 56]. Since H-NS and Sfx share the binding sites on the chromosome (Fig. 2C) and the preference for DNA topology (Fig. 3), we surmised that Sfx may also form bridged filaments. We first tested this idea *in vitro*, using an assay developed to observe bridging of two linear DNA fragments by H-NS [82]. As a probe, we chose the *bglG* gene, which has been used to study H-NS bridging [83]; both H-NS and Sfx bind to and silence the *bgl* operon (see below). This analysis shows that Sfx bridges DNA similarly to H-NS *in vitro* (Supplementary Fig. S4). We note that these assays were performed with the linear DNA, whereas our data suggest that Sfx DNA-binding (and possibly bridging) properties are affected by DNA topology.

Next, we wanted to assess the possibility that Sfx bridges DNA *in vivo*. Recent high-resolution micro-C chromosome conformation capture experiments [84] demonstrate that H-NS bridges stems of short (∼2–10 kb) chromosomal hairpins (CHINs). Mapping the CHIN coordinates to our H-NS ChIP-seq data shows that the CHIN stems correspond to peaks in ChIP profiles, whereas the CHIN loops form valleys; H-NS does not bind to CHIN loops, which are likely occupied by HU [84]. We carried out a genome-wide comparison of Sfx and H-NS occupancy in the CHIN regions (representative examples shown in Supplementary Fig. S5, Dataset 1E–K). Our analysis of the chromosomal ChIP signals of Sfx and H-NS reveals that in CHIN regions bound by both proteins, Sfx displays similar peaks but much shallower valleys (Supplementary Fig. S5B).

These results suggest that Sfx can bridge CHIN stems similarly to H-NS *in vivo* but, unlike H-NS, is able to spread into CHIN loops, possibly displacing loop-binding proteins. A potential caveat to this interpretation is that our ChIP-seq analysis of Sfx and H-NS binding to the chromosome was carried out in stationary cells, whereas the micro-C mapping was done in the exponential-phase cells [84]. However, comparison of our dataset to two published ChIP-seq datasets from the log-phase cells shows that H-NS binding is similar between the exponential and stationary phases, with Spearman correlation >0.8 (Supplementary Fig. S5A), and we are not aware of any published data showing that H-NS does not form short-range DNA bridges (CHINs) in the early stationary phase. Our planned micro-C probing will determine whether CHIN-like structures form on R6K and are stabilized by Sfx at different stages of cell growth.

### Sfx mimics H-NS effects mediated by DNA and RNA interactions

**Figure 4.**
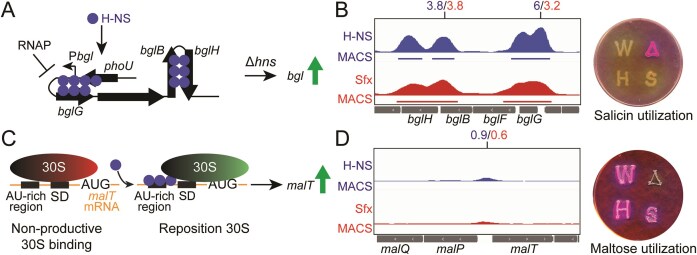
Sfx can complement the *hns* deletion phenotypes. (**A**) Mechanism of H-NS-mediated silencing of the *bgl* operon. Bridged H-NS filaments block transcription initiation and elongation. (**B, D**) Left, the tracks represent FE, which ranged from 0 to 7; example peak heights and MACS plots for statistically significant regions are indicated. Right, *in vivo* test results of sugar utilization using the MacConkey indicator agar. The *E. coli* ∆*hns* strain was transformed with plasmids expressing H-NS (**H**), Sfx (**S**), or an empty vector (∆); the WT *E. coli* (**W**) was transformed with an empty vector. Single colonies were patched onto MacConkey plates supplemented with 0.6% salicin or 0.4% maltose and incubated for 20 h at 30°C; a representative plate (out of five biological replicates tested) is shown. (**C**) Mechanism of H-NS-mediated activation of the *mal* operon. The *malT* mRNA contains a suboptimal Shine–Dalgarno (SD) sequence. H-NS binds to the AU-rich region of the *malT* mRNA and repositions the 30S from a non-productive state to an active translation initiation state, thereby increasing the translation of MalT, the activator of the *mal* operon transcription.

Our findings that Sfx and H-NS share many binding sites on the chromosome are consistent with a previous report that Sfx can at least partially complement some phenotypes of the ∆*hns* strain, such as motility and growth defects [24]. While H-NS has been extensively studied over the years, nothing is known about the molecular mechanism of Sfx. We thought that comparing the effects of Sfx and H-NS on genes where the mode of action of H-NS is known could shed light on that of Sfx. We first tested the ability of Sfx to silence the cryptic *bgl* operon, which enables *E. coli* to catabolize β-glucosides, the activity that can be assayed on MacConkey indicator agar supplemented with selected sugar. The *bgl* operon is one of the best-studied targets of H-NS, which binds to high-affinity DNA sites flanking the promoter and forms filaments that block both sense and antisense transcription (Fig. 4A) [11, 56, 85]. H-NS inhibits RNAP binding to the promoter [85] and increases Rho-dependent termination by inducing RNAP pausing during elongation [56]. The block to elongation requires the formation of bridged H-NS filaments [56] and is robustly stimulated by Hha and StpA, which form mixed filaments with H-NS [21]. Unlike H-NS-only filaments, the StpA-only filaments efficiently blocked RNAP progression [21], a finding of particular interest given that Sfx is a closer paralog to StpA [24]. Our results show that H-NS and Sfx have similar profile shapes on the *bglHGFB* genes with 3–6 fold apparent enrichment (Fig. 4B) and, when expressed from their native promoters (as described in [24]), can restore the *bgl* operon silencing in the *∆hns E. coli* strain, as assayed on MacConkey salicin plates (Fig. 4B).

Although most regulatory effects of H-NS are mediated through DNA interactions, H-NS and StpA bind RNA [86, 87] and can control gene expression post-transcriptionally. For example, H-NS and StpA activate expression of the *E. coli* maltose regulon by increasing translation of *malT*, a gene that encodes the activator of the maltose regulon [88, 89]. This effect was shown to depend on H-NS-mediated 30S repositioning (Fig. 4C) on the *malT* mRNA [88]. As expected, we observed that the *hns* deletion abolished maltose utilization (as assayed on MacConkey maltose plates; Fig. 4D). The ectopic expression of either *sfx* or *hns* restored maltose utilization, and neither protein exhibited enrichment on *malT* DNA (less than one-FE), suggesting that Sfx may be able to bind RNA.

To identify potential RNA ligands of Sfx, we used RNA ChIP-seq, in which the samples were treated with DNase after crosslinking reversal to enrich for Sfx-bound RNAs, which were then sequenced. This analysis identified several enriched RNAs, such as *ssrA* with four-FE, and we were able to confirm that Sfx interacts with the SsrA RNA *in vitro* (Supplementary Fig. S6, Dataset 1 LM). However, the physiological significance of this observation remains to be determined; e.g. we did not observe enrichment in *malT* mRNA, even though our complementation results (Fig. 4D) indirectly support a model in which Sfx binds RNA. It is possible that Sfx pulls down more abundant RNAs; calculated from our RNA-seq data, the *ssrA* RNA is ∼268 times more abundant than *malT* RNA. Another reason is that most of our RNA ChIP-seq reads are rRNA, which reduces the chance of detecting *malT* RNA.

### Sfx is preferentially localized to the R6K vir operon

Our results demonstrate that Sfx has minimal effects on chromosomal gene expression (Fig. 1F), likely due to being outcompeted by H-NS at most sites (Fig. 2C). However, the two proteins localize to similar AT-rich regions and recognize similar DNA motifs (Fig. 2E). This pattern is consistent with the ability of the ectopically expressed Sfx to substitute for missing H-NS, as shown here (Fig. 4) and in the previous report [24], and reminiscent of other plasmid-encoded H-NS homologs [27].

**Figure 5.**
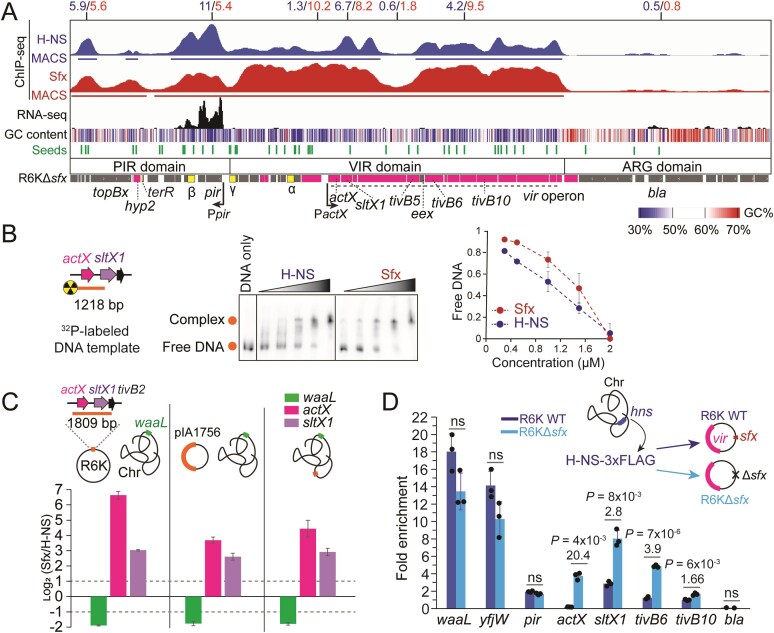
H-NS is largely excluded from the R6K VIR domain. (**A**) RNA seq track of WT R6K is shown along with H-NS and Sfx ChIP-seq tracks. Scale of H-NS and Sfx ChIP-seq tracks ranged from 0 to 12, with example peak heights indicated on top. MACS plot below ChIP-seq highlights statistically significant regions bound by H-NS (blue) or Sfx (red). The predicted high-affinity binding sites (seeds) are shown as green bars. The proposed three domains of R6K are indicated. The GC content, calculated in a 25-bp sliding window, is shown as a heatmap below the tracks. Significantly upregulated genes by *sfx* deletion are highlighted in magenta in the R6KΔ*sfx* map. The promoters of *pir* (P*pir*) and the *vir* operon (P*actX*) are indicated. The positions of three replication origins are shown in yellow, and the replication terminus (*terR*) in lime yellow. (**B**) Interactions of Sfx and H-NS with the *actX–sltX1* fragment were tested by EMSA. Protein concentrations ranged from 0.3 to 2 μM; the radioactively labeled DNA was fixed at 35 nM. The proportion of free DNA is plotted against protein concentrations; error bars are SD (*n* = 3). (**C**) Moving *actX–sltX1–tivB2* fragment from R6K into plasmid pIA1756 (Supplementary Fig. S8) and chromosome. ChIP-qPCR results show the enrichment of selected fragments. FE (ChIP/Input) was first calculated using Cq from qPCR, and the log2(Sfx FE/H-NS FE) is shown as bar graph. Error bars are SD (*n* = 3). (**D**) ChIP-qPCR quantifies the FE of H-NS on different genes. The FEs, calculated for both WT and Δ*sfx* R6K, are shown as bar graph. Error bars are SD (*n* = 3). A two-tailed *t*-test assuming unequal variances was used to calculate *P* values. Fold change (FC) was calculated as FE (R6KΔ*sfx*) / FE (WT R6K). Values for genes with *P* value < .05 and FC > 1.5 are indicated at the top of bars, and “ns” is indicated otherwise.

Our analysis of Sfx and H-NS binding to R6K reveals a strikingly distinct pattern. Sfx has ∼5-fold and H-NS has ∼11-FE on *pir* gene; but Sfx forms two continuous multi-kb peaks along the *vir* operon with ∼10-FE, whereas H-NS binding is reduced to less than four-FE in most areas (Fig. 5A, Dataset 1E–H), even though the *vir* operon is very AT-rich (Fig. 1A). Our ChIP-seq and RNA-seq data suggest that R6K is organized into three distinct domains (Fig. 5A). (i) The ARG domain, a transposition island that encodes three antibiotic resistance genes, is GC-rich and does not bind either H-NS or Sfx. (ii) The PIR domain, which contains the highly transcribed *pir* gene, has high H-NS peaks. Gene expression of the PIR domain is largely unaffected by Sfx; the only exception is *hyp2*, which is upregulated upon *sfx* deletion (Fig. 1F). This result, however, could be artefactual: in the R6K*∆sfx*, the *hyp2* gene is placed next to the *sfx* gene promoter (Fig. 5A, Dataset 2), located at the end of the *topBx* gene (which encodes a putative topoisomerase), and may become fortuitously activated. (iii) The VIR domain, which includes the *vir* operon and other conjugation genes, is almost continuously covered by Sfx. Consistently, the conjugation function-related VIR domain genes are silenced by Sfx (Fig. 1F).

The Sfx VIR footprint is demarcated by the main origin of R6K replication, γ *ori*, and the highly transcribed *pir* gene on one side, and by a “red” wall (> 65% GC, with a potential to form G-quadruplets) on the other side (Fig. 5A), features that could act as barriers to the Sfx filament extension. Notably, a gap in Sfx and H-NS occupancy occurs near the center of the VIR peak (Fig. 5A), in a region characterized by the typical AT-rich content of the *vir* operon (Fig. 1A). At least two mechanisms could explain this discontinuity in the Sfx filament. First, we identified potential high-affinity Sfx-binding sites on R6K using Fuzzy Search DNA [46] with the shared pattern AATAATAAATATAT as a query. Sequences with scores greater than or equal to 10 (score of 14 is a perfect match) were selected (Seeds in Fig. 5A). Each VIR domain peak contains many Seeds (Fig. 5A), whereas the valley only has two (Fig. 5A). If Sfx requires several Seeds to form stable filaments, it could be outcompeted by other proteins in Seed-poor regions. Second, we speculate that transertion, a process in which transcription, translation, and insertion of a protein into the membrane are closely coupled [90], anchors the VIR domain DNA to the cytoplasmic membrane, effectively partitioning this region into two Sfx-interacting VIR subdomains. Two genes located in/near the valley (Fig. 5A), *tivB5* and *eex*, are expected to undergo transertion. The *tivB5* gene encodes a putative adhesin that must traverse both cellular membranes *en route* to its location at the tip of the conjugative pilus [91]. The *eex* gene encodes a putative entry exclusion factor, a membrane lipoprotein. Interestingly, Sfx has only a modest effect on *tivB5* and *tivB6* expression and no effect on *eex* (Fig. 1F), suggesting that these genes are at least partially independent of Sfx control.

Sfx and H-NS recognize similar AT-rich motifs and, on the chromosome, H-NS peaks are more prominent than Sfx peaks (Fig. 2C–E); we did not observe a single instance of a higher Sfx peak. Although the H-NS/Sfx peak heights cannot be used to infer their relative binding affinities, the reversal of this pattern in the R6K VIR domain (and to a lesser extent in the PIR domain) (Fig. 5) implies the existence of mechanisms that mediate preferential recruitment of Sfx or selective exclusion of H-NS to/from R6K.

### VIR domain preferentially binds Sfx on supercoiled DNA

To explain how Sfx is selectively targeted to the VIR domain, we examined several possibilities. *First*, Sfx may be preferentially recruited to R6K *in cis*. However, the published data [24] and our results demonstrate that Sfx expressed *in trans* from its cognate promoter on a low copy number plasmid (Fig. 2B and Supplementary Fig. S2B) silences R6KΔ*sfx* conjugation, which refutes this explanation.

Second, the R6K DNA may contain modifications that inhibit H-NS, but not Sfx binding. This explanation appears farfetched since (i) R6K does not encode a putative DNA-modification enzyme and (ii) the modification would be restricted to the VIR domain. Nonetheless, we compared H-NS binding to plasmid DNA extracted from cells to PCR-amplified DNA (Supplementary Fig. S7) and did not observe any differences, excluding DNA modification as the mechanism of H-NS exclusion.

Third, it is possible that H-NS prefers to bind the chromosome and is present in insufficient quantities to cover R6K. This explanation appeared unlikely because H-NS peaks in the *pir* gene and the β *ori* are as high as on the chromosome (Fig. 5A), and R6K increases the total DNA content of the cell only by 14%, an increase that may be offset by autoregulation of H-NS [68].

Fourth, Sfx may bind the VIR domain with higher affinity than H-NS. Using a linear 1218 bp DNA fragment encompassing the *actX–sltX1* region, which is strongly associated with Sfx (Fig. 5A and B), we found that H-NS and Sfx bind DNA similarly (Fig. 5B). To test whether Sfx binding would be selectively favored on supercoiled DNA, we inserted the *actX–virB1* fragment into an unrelated 4 kb plasmid (pIA1756; Supplementary Fig. S8A) and assayed the H-NS and Sfx binding with ChIP-qPCR (the positions of probes are shown in Supplementary Fig. S8B). Our results reveal that the apparent enrichment of Sfx in the chromosomal *waaL* gene is less well than that of H-NS (Fig. 5C), in line with the ChIP-seq observations (Fig. 2D). In contrast, Sfx preferentially binds to the *actX* and *sltX1* genes on the plasmid (Fig. 5C). We next moved the fragment containing *actX* and *sltX1* genes to the chromosome, choosing the CP4-57 prophage region to which H-NS binds well, but Sfx does not (Fig. 2D); again, we found that Sfx binds *actX* and *sltX1* genes better than H-NS (Fig. 5C). These results demonstrate that Sfx preferentially binds to the VIR domain segment located on the negatively supercoiled DNA (either the chromosome or a plasmid), but not on the linear DNA.

We hypothesize that Sfx recognizes a specific structure that forms in the topologically constrained VIR domain. Recognizing that probing the VIR domain DNA structure in the cell would be challenging, we carried out two types of experiments that lend support to this hypothesis. First, we showed that the inhibition of DNA gyrase by novobiocin abolishes conjugation silencing by Sfx (Supplementary Fig. S3). *Second*, it has been shown that melting and the transition from B- to L-form DNA are sensitive to GC content [92, 93]. The 4 kb plasmid (pIA1756, 44% GC content) that contains the *actX–sltX1* fragment and selectively binds Sfx (Fig. 5C) displayed highly compact topologies in AFM images, as did a similarly sized control plasmid (pBR322, 54% GC) (Supplementary Fig. S8C), whereas a 5.8 kb plasmid pDM_N1_400 [94] (54% GC; a 1.5 kb fragment was inserted into the pBR322 backbone) shows a relaxed topology (Supplementary Fig. S8C, Supplementary Table S1). Ongoing single-molecule studies of larger segments of the VIR domain may reveal distinct topology.

Does this unusual structure block H-NS binding to the R6K VIR domain? To explore this possibility, we compared H-NS binding to selected genes on the WT R6K and R6KΔ*sfx* using ChIP-qPCR. As controls, we used the chromosomal *waaL* and *yfjW* genes, which are strongly bound by H-NS (Fig. 2D). We found that H-NS binding to the chromosome was not significantly affected by the *sfx* deletion on R6K, as could be expected. We also did not observe changes in H-NS association with the PIR and ARG domains of R6K (Fig. 5D), using the *pir* and *bla* genes as probes, respectively. In sharp contrast, H-NS binding to the *vir* operon was notably increased upon the deletion of *sfx* from R6K: the H-NS ChIP signal enrichment in *actX* increased 20 times, and 3–4 times for *sltX1* and *tivB6* genes (Fig. 5D). We conclude that the selective H-NS exclusion from the R6K VIR domain is mediated by Sfx/DNA complexes, which assemble on the negatively supercoiled DNA.

### Phase separation promotes H-NS exclusion from R6K

**Figure 6.**
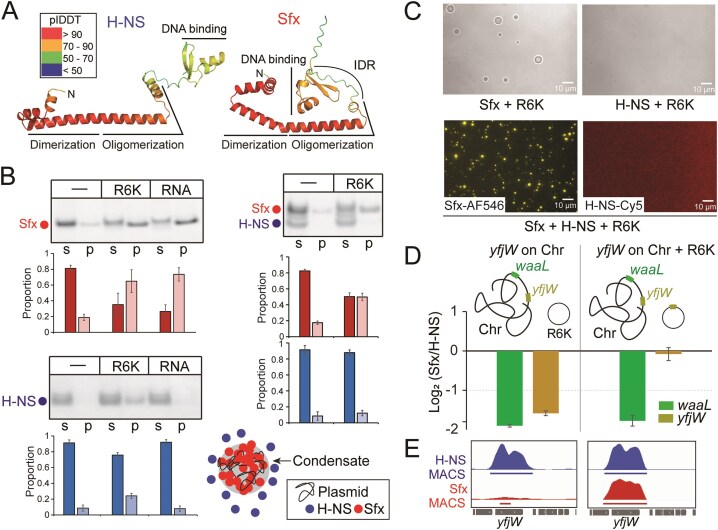
Phase separation of Sfx and R6K. (**A**) Structural models of Sfx and H-NS were predicted by AlphaFold3. (**B**) Pelleting assays of Sfx and H-NS with R6K or RNA. (Left), Sfx and H-NS were tested separately. The RNA is a total RNA extracted from the exponential phase of *E. coli* cells. Each protein (at 2 μM) was pelleted with 5 ng/μl R6K or 30 ng/μl total RNA. s, supernatant. p, pellet. (Right) Sfx and H-NS were mixed. Bar graphs (Sfx, red; H-NS, blue with the darker shade corresponding to the soluble fraction) were generated from three biological repeats; error bars are the SD. (**C**) Microscopy of Sfx phase separation with R6K. In the presence of 3% PEG8000, 2 μM unlabeled proteins were mixed with 0.5 μM fluorescently labeled proteins and 5 ng/μl R6K. (Top) Sfx and H-NS are tested individually. Bright-field views are shown. (Bottom) Sfx and H-NS are mixed. Fluorescence views were obtained for AF546-labeled Sfx and Cy5-labeled H-NS. Scale bar, 10 μm. (**D**) ChIP-qPCR results of the *yfjW* gene enrichment after moving it into R6K. FE (ChIP/Input) was first calculated using Cq from qPCR, and then the log_2_(Sfx FE/H-NS FE) is shown as bar graph. Error bars are SD (*n* = 3). (**E**) ChIP-seq tracks of the *yfjW* gene. Left, ChIP-seq with *yfjW* on the chromosome. Right, ChIP-seq with *yfjW* on the chromosome and R6K. Peaks represent FE, and the same scale is used for both H-NS and Sfx tracks within each scenario. MACS plot highlights statistically significant regions bound by H-NS (blue) or Sfx (red).

Sfx is predicted to be folded similarly to H-NS and to contain dimerization, oligomerization, and DNA-binding modules, but their length and orientation may differ (Fig. 6A). R6K Sfx and ∼73% of Sfx homologs from conjugative IncX plasmids have predicted IDRs, whereas *E. coli* H-NS does not (Figs 1B and 6A). IDRs are often involved in phase separation [95], a phenomenon that creates membrane-less organelles in cells [96]. We wondered whether phase separation can be utilized to exclude H-NS from Sfx-R6K. We first used a pelleting assay to examine whether Sfx can form phase-separated condensates. We found that purified Sfx can be efficiently pelleted with R6K or total RNA (Fig. 6B), whereas H-NS, less efficiently, is pelleted with R6K in the absence (Fig. 6B, left), but not in the presence of Sfx (Fig. 6B, right), supporting the idea that Sfx outcompetes H-NS for binding to R6K. These results can be explained by macromolecular aggregation or phase separation.

To evaluate the possibility of phase separation, we utilized microscopy. From bright-field microscopy, we find that Sfx forms condensates with R6K and RNAs, while in the same conditions, HNS does not (Fig. 6C, top and Supplementary Fig. S9A). Next, we asked if H-NS can enter the Sfx–R6K condensate. Pelleting assays show that when Sfx, H-NS, and R6K are present together, Sfx is pelleted while H-NS remains mostly in the supernatant (Fig. 6B); 300 mM KCl, but not 5% 1,6-hexanediol, disrupts the formation of the Sfx–R6K pellet (Supplementary Fig. S9B), indicating that the assembly is mainly driven by electrostatic interactions. The fluorescence microscopy analysis of samples containing AF546-labeled Sfx, Cy5-labeled H-NS, and R6K shows that Sfx is concentrated inside droplets, whereas H-NS is evenly distributed (Fig. 6C, bottom). However, H-NS does not appear to be selectively excluded from Sfx–R6K condensates (Fig. 6C), consistent with the ChIP-seq data showing that H-NS weakly binds to the VIR domain (Fig. 5A).

These *in vitro* assays suggest that phase separation could assist Sfx in safeguarding the R6K VIR domain from nonproductive (in terms of gene expression silencing) association with H-NS indirectly, by sequestering Sfx within a microcompartment formed by up to 16 copies of R6K. If this compartmentalization occurs, Sfx should possess a competitive binding advantage for any gene located on the R6K plasmid. To test this, we used the *yfjW* gene from the CP4-57 prophage, which exhibits the highest chromosomal H-NS occupancy and minimal Sfx binding, indicating that Sfx binding to *yfjW* is outcompeted by H-NS (Fig. 2D). We postulated that moving the *yfjW* gene to R6K should favor binding of Sfx. We used ChIP-qPCR to compare two scenarios (i) *yfjW* on the chromosome only, and (ii) *yfjW* present on both the chromosome and within the R6K ARG domain (Fig. 6D). We found that while the relative binding of H-NS and Sfx to the *waaL* gene remained the same across scenarios, Sfx recruitment to *yfjW* shifted significantly. The relative FE of Sfx over H-NS, log_2_(Sfx/H-NS), at *yfjW* increased from −1.6 to −0.08 in the second scenario (Fig. 6D). This shift was confirmed by ChIP-seq analysis, demonstrating that Sfx efficiently binds *yfjW* only on R6K (Fig. 6E, Dataset 1 NO) and suggesting that Sfx possesses a localized competitive advantage within the R6K context.

## Discussion

Many bacteria utilize two (or more) paralogous histone-like proteins to silence horizontally transferred genes. Examples include H-NS and StpA in *E. coli*, MvaT and MvaU in *Pseudomonas aeruginosa*, and Lsr2 and LsrL in Actinobacteria [10]. The secondary protein (StpA, MvaU, LsrL) may appear partially redundant, having fewer targets than the major silencer [65, 97–101]. In addition, hundreds of H-NS homologs have been identified on plasmids [102]. It remains unclear how these silencers coordinate or if the “redundant” paralogs possess specialized functions.

Here, we examined an interplay between H-NS and its homolog, Sfx, which is encoded on R6K plasmid and serves to suppress its conjugation. A recent report highlights a striking functional asymmetry: while Sfx can replace chromosomal H-NS functions, H-NS is incapable of silencing R6K transfer [24]. Our ChIP-seq analysis reveals that, on the chromosome, Sfx and H-NS binding sites largely overlap; both proteins recognize similar DNA motifs (Fig. 2C and E) and have an apparent preference for the negatively supercoiled DNA (Fig. 3). However, Sfx is outcompeted by H-NS at most sites (Fig. 2C and D) and, consistently, Sfx deletion has little effect on the chromosomal gene expression (Fig. 1F). On R6K, this pattern is drastically different: Sfx is strongly (∼10-fold) enriched across the entire VIR domain, which includes the *vir* operon, whereas H-NS is largely outcompeted by Sfx (Fig. 5A). Curiously, the peak heights of Sfx and H-NS in the PIR domain are comparable (Fig. 5A). Given that similar apparent enrichment for both proteins occurs near the chromosomal origin and terminus (Fig. 2C), we hypothesize that the γ origin and replication terminus (*terR*) of R6K [103, 104] may likewise dictate these patterns.

### Hypothetical mechanisms of preferential Sfx targeting to R6K

**Figure 7.**
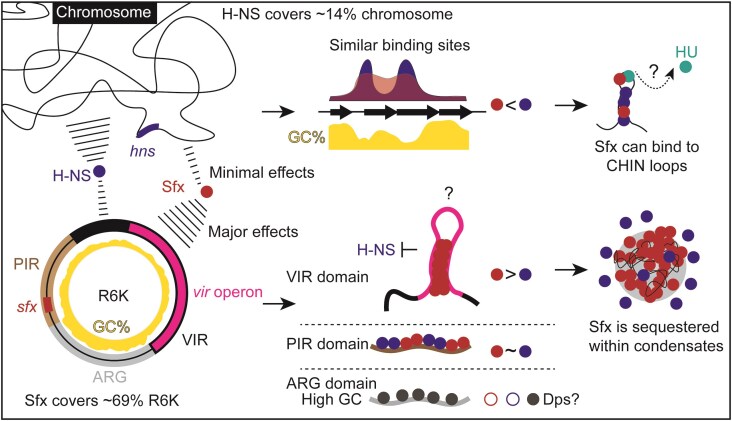
The proposed model of selective targeting of Sfx to the R6K conjugal transfer genes. While Sfx strongly silences the R6K *vir* operon, H-NS exerts minimal influence, even when Sfx is absent. Although H-NS and Sfx occupy similar chromosomal sites, Sfx uniquely extends into CHIN loops and could displace proteins bound therein. Our analysis of the R6K plasmid reveals three distinct domains that differ in their binding to H-NS and Sfx. The PIR domain binds both proteins, while the ARG domain binds neither, potentially using Dps to fill the void. We propose that H-NS is excluded from the VIR domain through two primary drivers: the inherent DNA structure of the *vir* operon, which promotes the formation of stable Sfx filaments, and the phase separation of Sfx-R6K complexes. See text for additional details.

Our data support two mutually reinforcing mechanisms that explain the observed selectivity of Sfx (Fig. 7). First, we hypothesize that the *vir* DNA forms a unique architecture recognized by Sfx as a high-affinity loading site, followed by the spread of Sfx filaments along the DNA, a shared behavior of H-NS homologs [10, 71, 72]. We demonstrate that Sfx cellular activity depends entirely on negative DNA supercoiling (Fig. 3 and Supplementary Fig. S3). Single-molecule [105] and high-resolution chromosome conformation capture [84] experiments demonstrate that H-NS pins plectonemes *in vitro* and *in vivo*, bridging CHIN stems [84]. Our ChIP-seq data reveal a distinct binding pattern for Sfx: while H-NS tracks with CHIN stems and loops (peaks and valleys), Sfx forms uniform, wider peaks across the entire hairpin (Fig. 2G and Supplementary Fig. S5B).

Single-molecule experiments on synthetic DNAs show that AT-rich sequences pin plectonemes at specific locations [106] to determine the overall DNA structure. Modeling indicated that DNA deformability, which can be calculated from dinucleotide steps, rather than overall AT content, underlies this behavior. The *vir* operon has a high AT content and many deformable dinucleotide steps, favoring conformational flexibility, and *vir* DNA may form compact structures (Supplementary Fig. S8C). Future experiments will determine the location and structure of the high-affinity Sfx binding site(s) that we propose to mediate its initial loading on R6K (Fig. 5A).

Second, we hypothesize that phase separation ensures that Sfx is sequestered within R6K condensates, promoting initial Sfx binding and spreading along the VIR domain and preventing Sfx “escape” to the chromosome, which has an excess of Sfx binding motifs. The Sfx sequestration would be expected to favor its binding to R6K. Indeed, Sfx binds better to the *actX* gene on R6K as compared to the same region placed on the chromosome or an unrelated plasmid (Fig. 5C). Strikingly, Sfx gains the ability to bind the *yfjW* gene, which shows no Sfx occupancy on the chromosome (Fig. 2D), once *yfjW* is moved to R6K (Fig. 6D and E). This sequestration will further bolster the competitive advantage of Sfx over H-NS, indirectly excluding H-NS from the preformed Sfx/DNA complexes. Pelleting and microscopy assays show that Sfx forms condensates with RNA and DNA (Fig. 6B and C; Supplementary Fig. S9). While H-NS can also phase separate at high concentrations, as shown by others [107], Sfx does so more readily. We considered the possibility that an IDR present in Sfx but not in H-NS (Fig. 6A) may promote phase separation. Our pelleting assay shows that replacing Sfx IDR with H-NS linker only moderately reduced pellet formation, whereas replacing Sfx CTD with H-NS CTD led to a larger reduction (Supplementary Fig. S9C). The molecular determinants of Sfx phase separation will be explored in future experiments. While (to our knowledge) phase separation has not been reported for the Sfx-homolog StpA, it also possesses a large degree of intrinsic disorder, and its weak and dynamic contacts with RNA are thought to underpin its ability to act as an RNA chaperone [108].

### Structural organization and regulation of R6K

R6K consists of three modules: a GC-rich ARG domain flanked by the AT-rich PIR and VIR domains (Fig. 7). The ARG domain does not bind either H-NS or Sfx (Fig. 5A); we hypothesize that this region is bound by Dps, the most abundant nucleoid-associated protein in the stationary phase [109], whose occupancy on the chromosomal DNA [110] is inversely correlated with that of H-NS (Supplementary Fig. S10). The PIR domain binds both Sfx and H-NS, whereas the VIR domain is almost continuously covered by Sfx and largely lacks H-NS (Fig. 5A). Our ChIP-seq data suggest that the VIR domain forms a plectoneme; Sfx occupies and likely bridges the plectoneme stems but does not bind to the loop region (Fig. 5A), which may be anchored to the membrane through transertion (see *Sfx is preferentially localized to R6K vir operon*).

In our model (Fig. 7), Sfx represses R6K conjugation directly, by roadblocking the transcribing RNAP, and indirectly, by excluding H-NS from the transfer operon. The second mechanism may be unexpectedly critical, given that H-NS, the major xenogeneic silencer known to target numerous foreign genes, is unable to silence R6K [24] for yet-unknown reasons. While many other DNA-binding proteins have been implicated in the silencing of AT-rich xenogenes in *E. coli* [111], none appears able to substitute for Sfx, whose loss leads to ∼1000-fold increase in R6K conjugation [24].

The multimodal silencing may be crucial for R6K, and in turn the host cell, because the loss of silencing filaments even on a single plasmid copy would lead to leaky expression of the T4SS genes, rendering the host vulnerable to pilus-specific phages. Accordingly, all 16 copies of R6K must be coordinately controlled by Sfx to ensure that conjugation is fully silenced. We propose that, in addition to conventional silencing of RNA synthesis, Sfx brings all R6K copies together by cross-bridging different plasmid molecules and promoting the formation of membrane-less cellular organelles. This multiprong strategy renders R6K self-sufficient and ensures its maintenance in diverse hosts, from *E. coli* to *Pseudomonas* [112], whose resident chromosomal silencers may fail to efficiently repress the conjugal transfer genes, as is the case with H-NS.

### Uninterrupted nucleoprotein filaments may be required to silence R6K conjugation

We posit that the efficient silencing of conjugation requires 100% occupancy of the *vir* operon by Sfx-only or mixed Sfx/H-NS filaments (Fig. 5A). The presence of mixed filaments is suggested by the H-NS ChIP signals observed within the continuous Sfx footprint. However, the relative levels of H-NS in these hypothetical mixed filaments may be lower than appear from the ChIP-seq peaks (see earlier for the discussion of ChIP efficiency).

Does the cellular concentration of Sfx suffice to fully cover the *vir* operon? Based on our ChIP-seq data, we estimate the potential binding areas for H-NS and Sfx as ∼0.6 Mb on the chromosome (primarily H-NS bound) and ∼0.4 Mb on R6K (the aggregate of 16 copies), respectively (Supplementary Fig. S2D). Our findings align with previous reports that *E. coli* H-NS covers 10%–15% (0.46–0.7 Mb) of the chromosome [113]. Based on *in vitro* measurements, one H-NS dimer is expected to cover ∼15 bp of DNA [83]. Assuming that Sfx makes similar contacts to DNA, ∼29 000 Sfx dimers would be needed to saturate 0.4 Mb of R6K DNA. While direct absolute measurements for Sfx are unavailable, quantitative proteomics shows that H-NS is present at 22 000 copies in the stationary phase *E. coli* [114]. Since Sfx is present in at least a 3.5-fold excess (Supplementary Fig. S2C), the total count of Sfx dimers exceeds 38 000 per cell. This abundance is more than sufficient to provide complete coverage of R6K, with a surplus of dimers available for binding to the chromosome.

We show that Sfx acts synergistically with Rho to block the expression of the R6K conjugal transfer *vir* operon during RNA chain elongation but does not inhibit initiation at the major *vir* operon promoter (Fig. 1D and E). Although this finding was unexpected given that Sfx-like proteins have been shown to act at the promoter [22, 50], the post-initiation action of Sfx is consistent with its ability to form bridged filaments (Supplementary Fig. S4), which can efficiently block the elongating RNAP [56]. Our ChIP-seq data show that, compared to H-NS, Sfx forms wider peaks along the same DNA regions (Fig. 2F and G; Supplementary Fig. S5B). It is plausible that Sfx forms longer, more stable filaments, which may displace other chromatin-associated proteins from the DNA, such as HU thought to bind CHIN loops in the log phase cells [84].

Differences in filament structure could explain why H-NS does not inhibit conjugation even though it binds the *vir* operon in the absence of Sfx (Fig. 5D): a break in the filament may enable recruitment of RNAP or accessory proteins and destabilize the filament further. A precedent for this exists in *Shigella* VirB, which binds to and spreads on DNA, removing negative supercoils and abrogating H-NS silencing [75]. Curiously, VirB is a member of the ParB family of DNA partitioning proteins, and R6K encodes a distant homolog of ParB.

While H-NS forms bridged filaments under some conditions, this property is promoted by Hha (H-NS-associated protein), a small (∼8 kDa) co-regulator that forms mixed filaments with H-NS [21, 115]. Hha enhances the ability of H-NS to arrest the elongating RNAP [20, 21] and increases the Sfx-mediated silencing of R6K conjugation [24]. To investigate Hha’s influence on Sfx–R6K binding, we performed ChIP-seq in an *hha* deletion strain. We observed that while the binding positions of Sfx on both R6K and the chromosome remained unchanged (Supplementary Fig. S11A and B), the peak heights on R6K were noticeably reduced in the absence of Hha (Supplementary Fig. S11, Dataset 1 PQ). These findings suggest that Hha and Sfx may co-assemble into mixed bridged filaments to achieve more potent transcriptional silencing. Consistently, Hha is pelleted together with Sfx (Supplementary Fig. S11D) at a 1:1 ratio, as observed with the H-NS/Hha complex [115]. Sfx paralog StpA also forms bridged filaments that efficiently block the RNAP progression, at least *in vitro* [21]. Modeling by AlphaFold 3 [116] shows that StpA and Sfx form similar oligomeric structures, while H-NS does not (Supplementary Fig. S12).

Plasmids employ sophisticated “stealth” mechanisms to mitigate host fitness costs. The H-NS homolog Sfh (pSfR27) serves as a classic molecular backup for H-NS, filling the chromosomal gaps in H-NS coverage caused by its repositioning to pSfR27 [26, 27], and R6K Sfx can also act as a molecular mimic of H-NS (Fig. 4). However, our findings reveal that Sfx utilizes a superior stealth strategy to combine near-zero impact on host gene expression with very potent silencing of the plasmid genes. Sfx clusters with R6K to silence the plasmid genes and reduce its off-target binding to the chromosome, while preventing H-NS dilution from the host genome and non-productive interactions with R6K. This precision targeting avoids the pitfalls of molecular mimicry, effectively neutralizing the fitness costs of plasmid carriage.

## Supplementary Material

gkag583_Supplemental_Files

## Data Availability

All plasmids and strains are available upon request. The NGS data generated in this study are deposited at the Gene Expression Omnibus with accession numbers GSE319657 for RNA-seq data and GSE319658 for ChIP-seq data. Psoralen crosslinking microarray data (GSE77687) collected at the stationary phase [78], GapR ChIP-seq (GSE152880) at the log phase [79], HupB ChIP-seq (GSE181767) at the log phase, HupB ChIP-seq (SRP008538) at the stationary phase [117], H-NS-3xFLAG ChIP-seq (GSE51582) at the log phase [118], tagless H-NS ChIP-seq at the log phase (GSE157512), and Dps ChIP-seq (GSE293552) at the stationary phase (24 h samples) [110] are available from NCBI.
