# Supplementary material for: Selective targeting of a histone-like silencer Sfx to the R6K conjugal transfer operon
Source: Nucleic Acids Res. 2026 Jun 10;54(11):gkag583. doi: 10.1093/nar/gkag583 (PMC13250579; doi:10.1093/nar/gkag583)
Supplement: gkag583_Supplemental_Files [file gkag583_supplemental_files.zip › Dataset legend 2.docx]

**Dataset 1**: (**A**) Sfx distribution analysis in IncX group plasmids. (**B**) Differential gene expression analysis result of the RNA-seq data. (**C**) MACS3 peak calling output: RNAP ChIP-seq repeat 1. False discovery rate (q-value) cutoffs are indicated in all MAS3 peak calling outputs. (**D**) MACS3 peak calling output: RNAP ChIP-seq repeat 2. (**E**) MACS3 peak calling output: H-NS-3xFLAG ChIP-seq repeat 1. (**F**) MACS3 peak calling output: H-NS-3xFLAG ChIP-seq repeat 2. (**G**) MACS3 peak calling output: Sfx-FLAG in wild-type E. coli ChIP-seq repeat 1. (**H**) MACS3 peak calling output: Sfx-FLAG in wild-type *E. coli* ChIP-seq repeat 2. (**I**) MACS3 peak calling output: HupB ChIP-seq at log phase repeat 1 (GSE181767). (**J**) MACS3 peak calling output: HupB ChIP-seq at log phase repeat 2 (GSE181767). (**K**) MACS3 peak calling output: HupB ChIP-seq at stationary phase (SRP008538). (**L**) MACS3 peak calling output: RNA ChIP-seq repeat 1. (**M**) MACS3 peak calling output: RNA ChIP-seq repeat 2. (**N**) MACS3 peak calling output: H-NS-3xFLAG in *E. coli* harboring pIA1710 (**Dataset 2**) ChIP-seq. (**O**) MACS3 peak calling output: Sfx-FLAG in *E. coli* harboring pIA1710 (**Dataset 2**) ChIP-seq. (**P**) MACS3 peak calling output: Sfx-FLAG in *E. coli* Δ*hha* ChIP-seq repeat 1. (**Q**) MACS3 peak calling output: Sfx-FLAG in *E. coli* Δ*hha* ChIP-seq repeat 2.

**Dataset 2**: GenBank format files of plasmid maps used in this study. The description of each plasmid is included in **Supplementary Table S1**.
