# Supplementary material for: Selective targeting of a histone-like silencer Sfx to the R6K conjugal transfer operon
Source: Nucleic Acids Res. 2026 Jun 10;54(11):gkag583. doi: 10.1093/nar/gkag583 (PMC13250579; doi:10.1093/nar/gkag583)
Supplement: gkag583_Supplemental_Files [file gkag583_supplemental_files.zip › Wang_Sfx_SI_R2.pdf]

Supplementary data

Selective targeting of a histone-like silencer Sfx to the R6K conjugal transfer operon

Bing Wang[1,2], Ritika Gupta[3], Nathan Blaine[1,4], Barbare Khitiri[1], Catherine Jordan[5], Natalia Molotievskiy[1], David Dunlap[3], Laura Finzi[3,5,6,7], and Irina Artsimovitch[1,2*]

[1]The Department of Microbiology and [2]The Center for RNA Biology, The Ohio State University, Columbus, OH 43210, USA

[3]Department of Physics and Astronomy, [5]Department of Bioengineering, [6]Medical Biophysics Program, and [7]Institute for Human Genetics, Clemson University, Clemson, SC 29634, USA

[4]Department of Chemical and Biological Science and Engineering, United States Military Academy, West Point, NY 10966, USA

*Correspondence to: artsimovitch.1@osu.edu

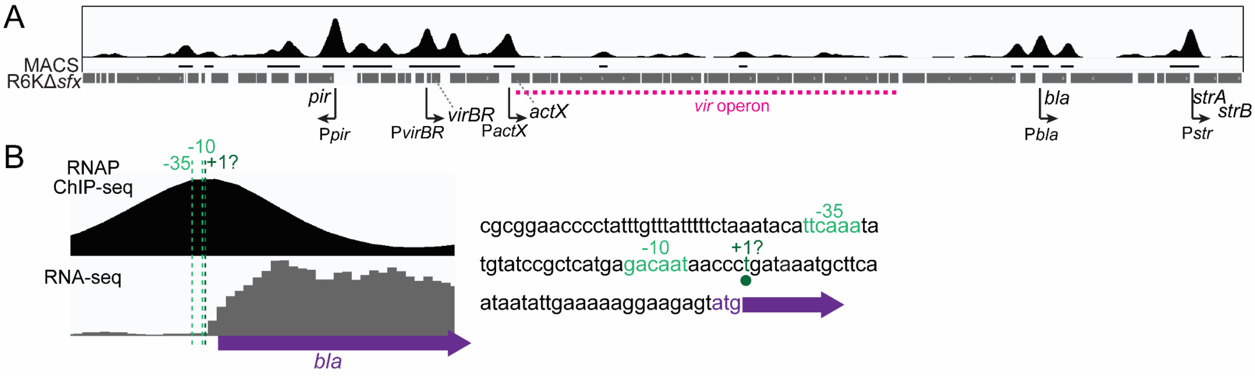

Supplementary Figure S1. RNAP ChIP-seq maps promoters in R6K. (**A**) The cell cultures harboring R6KΔ*sfx* were treated with rifampicin to enrich for promoter-bound RNAPs. The IGV tracks of two biological repeats, showing the fold enrichment (ranging from 0 to 10; FE = ChIP / Input), are displayed. MACS plot highlights statistically significant bound regions. All identified RNAP-binding peaks are in **Dataset 1CD**. The *pir* gene promoter has the highest RNAP peak, suggesting strong transcription. The *virBR* gene is co-transcribed with *hyp12* (see the p103 map in **Dataset 2**). The *bla* gene promoter overlaps with the known promoter region. The other two antibiotic resistance genes, *strA* and *strB*, are transcribed from the same promoter. No significant RNAP signals were detected inside the *vir* operon. (**B**) The published (1) *bla* promoter -10 and -35 elements are located around the RNAP ChIP-seq peak. The transcription start site is predicted by BDGP (2). RNA-seq coverage smoothed in 20-bp windows agrees with the locations of the promoter.

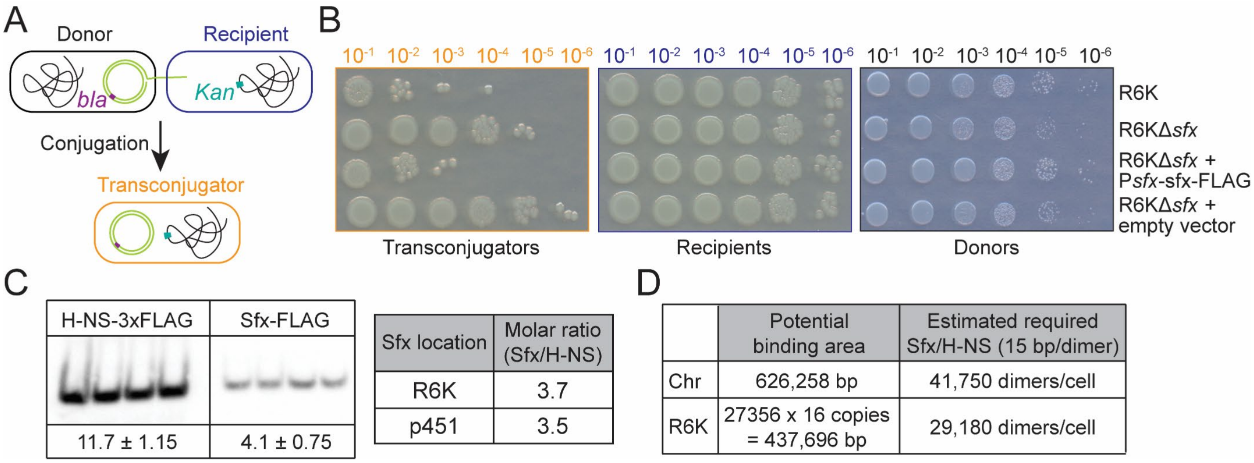

Supplementary Figure S2. Testing the activity and abundance of FLAG-tagged Sfx. (**A**) Schematic of conjugation assay. (**B**) Sfx-FLAG can inhibit conjugation *in trans*. (**C**) Determine H-NS/Sfx molar ratio by Western blot. For Sfx-FLAG located in R6K, *E. coli* MG1655 carrying R6K75*sfx*-FLAG (pIA1721; see **Supplementary Table S1** for plasmids) and *E. coli* MG1655 *hns*-3xFLAG carrying R6K75 (pIA1682) were used. For Sfx-FLAG located in p451, *E. coli* MG1655 carrying R6KΔ*sfx/*p451 and *E. coli* MG1655 *hns*-3xFLAG carrying R6KΔ*sfx/*p452 were used. Blot images of four biological repeats of Sfx-FLAG expressed from p451 are shown. The data (mean ± SD) represent the relative abundance of the target protein. The Western blot signal is normalized by the total input protein for each lane, quantified by Coomassie Blue staining of the SDS-PAGE gel. According to the technical notes from Millipore Sigma, 3xFLAG has at least ten times the detection sensitivity of a single FLAG. Thus, we converted the relative abundance by a factor of ten to obtain the molar ratio of Sfx/H-NS. When Sfx-FLAG was expressed from p451, Sfx/H-NS = 3.5 [4.1 x 10 / 11.7]. The same analysis was done with Sfx-FLAG expressed from its native locus in R6K, yielding Sfx/H-NS = 3.7. (**D**) Potential binding areas on the chromosome and R6K are estimated from the binding peaks of H-NS on the chromosome and binding peaks of Sfx on R6K, respectively. The required numbers of Sfx and H-NS dimers are estimated assuming that both proteins cover ~15 bp of DNA/dimer (3).

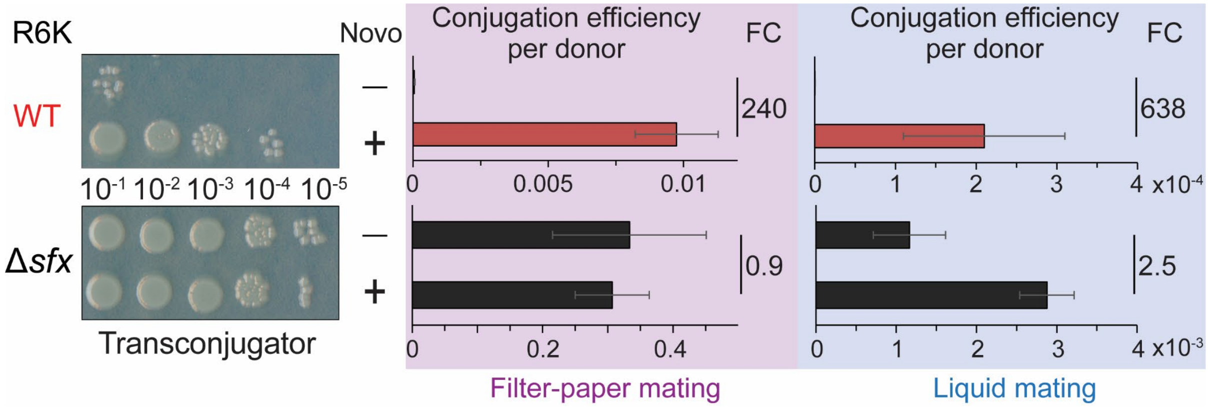

Supplementary Figure S3. Effects of novobiocin (Novo) on R6K conjugation. Representative plates from four independent biological repeats are shown for WT (wild-type) and Δ*sfx* (*sfx* deletion R6K), which visualize the transconjugator in the filter-paper mating assay. Serial dilutions are indicated. Conjugation efficiency per donor is shown as bar graphs. Error bars represent SD (n = 4). Fold of change (FC) = novo(+) / novo(-).

Hundreds of fold increase in conjugation efficiency is observed for WT R6K in both mating assays, whereas small or no changes are seen for R6KΔ*sfx.*

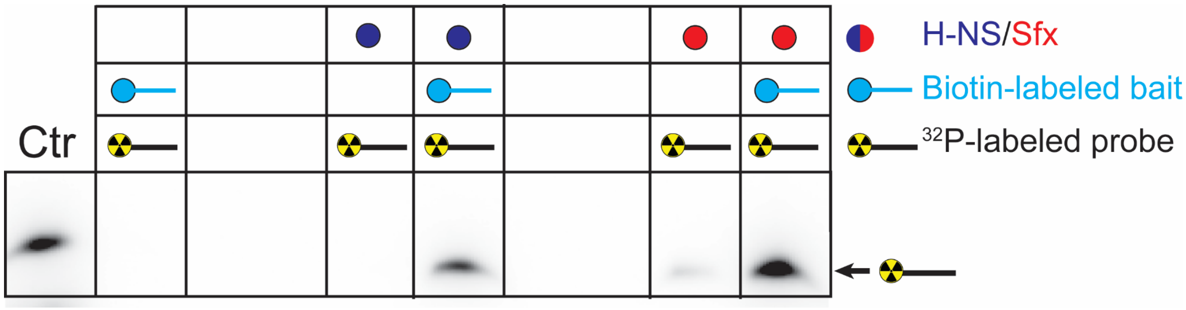

Supplementary Figure S4. Sfx forms bridged DNA complexes. The assay was adapted from the H-NS bridging assay (4). The DNA bait and probe from *bglG* gene, which has been used for H-NS bridging assay (3), were made by PCR (**Supplementary Table S1**). The DNA bait was biotinylated, and the DNA probe was radioactively labeled. For each reaction, 2 µl Dynabeads MyOne Streptavidin T1 (ThermoFisher, cat# 65601) were washed once with 50 µl of 1x PBS (Sigma, cat# P4417), and twice with 100 µl of Coupling Buffer2 (CB2: 20 mM Tris-HCl, pH 8.0, 1 mM EDTA, 0.5 M NaCl, 0.1 % Tween20). After washing, the beads were resuspended in 10 µl CB2 containing 100 nM biotinylated DNA bait. The bead suspensions were incubated for 30 min on a rotary shaker (1000 rpm) at 25 °C. After incubation, the beads were washed twice with 200 µl Incubation Buffer3 (IB3: 10 mM Tris-HCl, pH 8.0, 50 mM KCl, 10 mM $MgCl_2$, 0.1 mg/ml BSA (NEB, cat# B9000), 0.1% Tween 20, and 5 mM 2-mercaptoethanol) before resuspension in 9 µl IB3 containing 20 nM radioactive DNA probe. Finally, 1 µl H-NS or Sfx was (where indicated) added to a final concentration of 5 µM, and the mixture was incubated for 30 min on a shaker (1000 rpm) at 25 °C. The beads were washed twice with 200 µl IB3 to remove unbridged DNA probe before resuspension in 12 µl Bridge Stop Buffer (10 mM Tris-HCl, pH 8.0, 0.25 % SDS, 5 mM EDTA, 3 % glycerol, 0.02 % Orange G, and 500 mM NaCl). The bridged probe was resolved on a 3% polyacrylamide gel in 0.5x Tris-borate-EDTA (TBE) buffer. Ctr, the probe was loaded to mark its position on the gel. The gels were imaged by Amersham Typhoon 5.

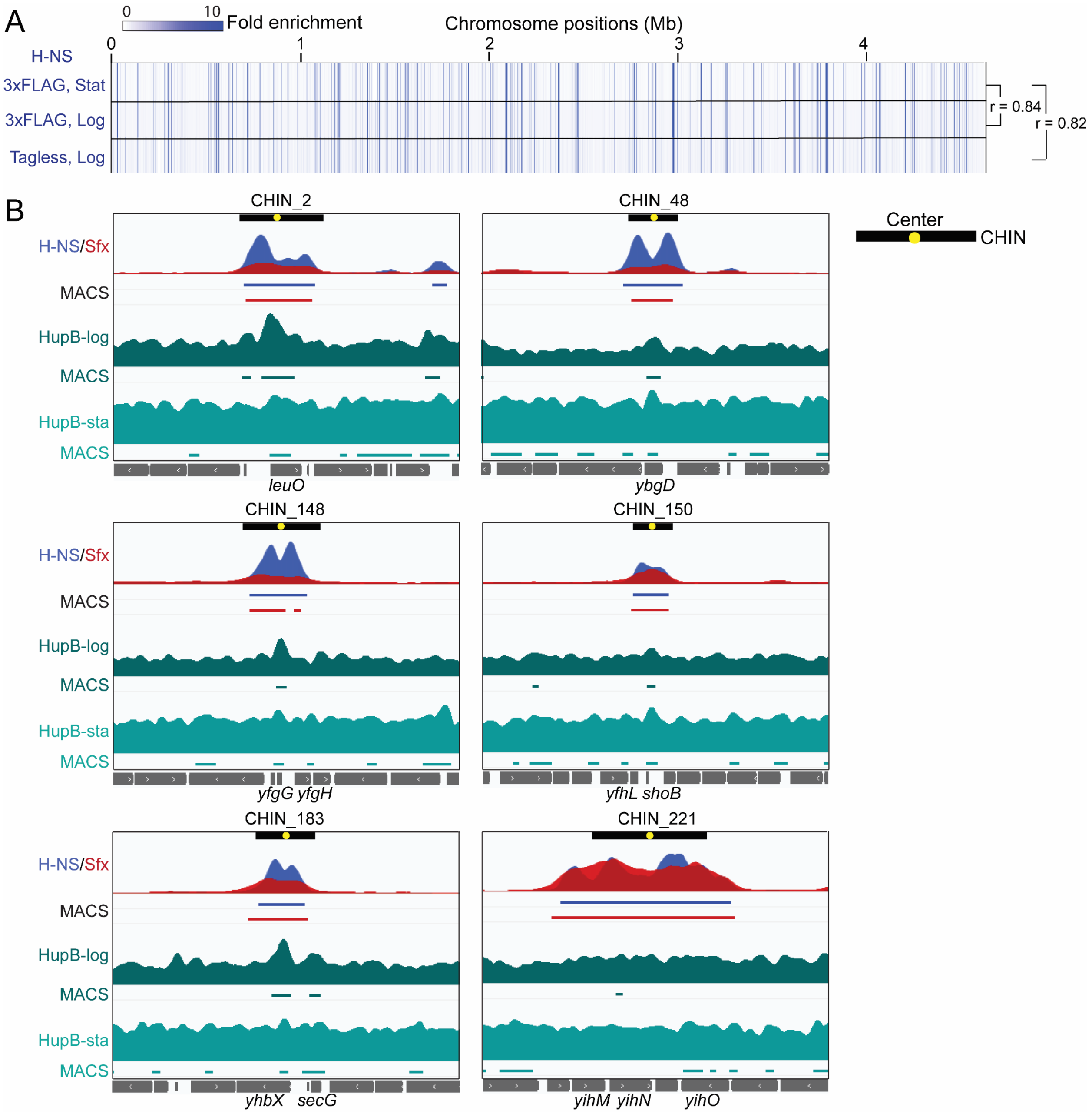

Supplementary Figure S5. Sfx can form continuous peaks through the CHIN loops. (**A**) Heatmap tracks of H-NS binding profiles on the chromosome. Spearman correlation (r) is calculated by binning reads at 300 bp. 3xFLAG, Stat: H-NS-3xFLAG profiles collected in this study. 3xFLAG, Log: HNS-3xFLAG profiles at log phase (GSE51582) (5). Tagless, Log: tagless H-NS profile in mid-log phase (GSE157512). (**B**) ChIP-seq tracks show examples of CHINs. CHIN coordinates from Gavrilov *et al*. (6) are mapped to H-NS and Sfx ChIP-seq profiles. CHIN centers (loops) are located in the valley of H-NS binding peaks. Below the H-NS/Sfx ChIP-seq

track, HupB ChIP-seq tracks from the log (HupB-log) and the stationary (HupB-sta) phase are shown. We note that the background noise cannot be confidently removed from the HupB peaks, because 1) the peaks were called without a control sample, and 2) HupB-sta has only one biological repeat. H-NS/Sfx tracks represent FE in the range of 0 to 8. HupB tracks are pileup read counts: HupB-log, ranged from 0 to 450; HupB-sta, 0 to 2500. MACS plot highlights statistically significant bound regions by H-NS (blue), Sfx (red), HupB-log (dark cyan), and HupB-sta (light cyan) (**Dataset 1E-K**). Sfx extends through the H-NS peak valley, suggesting Sfx may compete with HU for binding the CHIN loops (6); see main text.

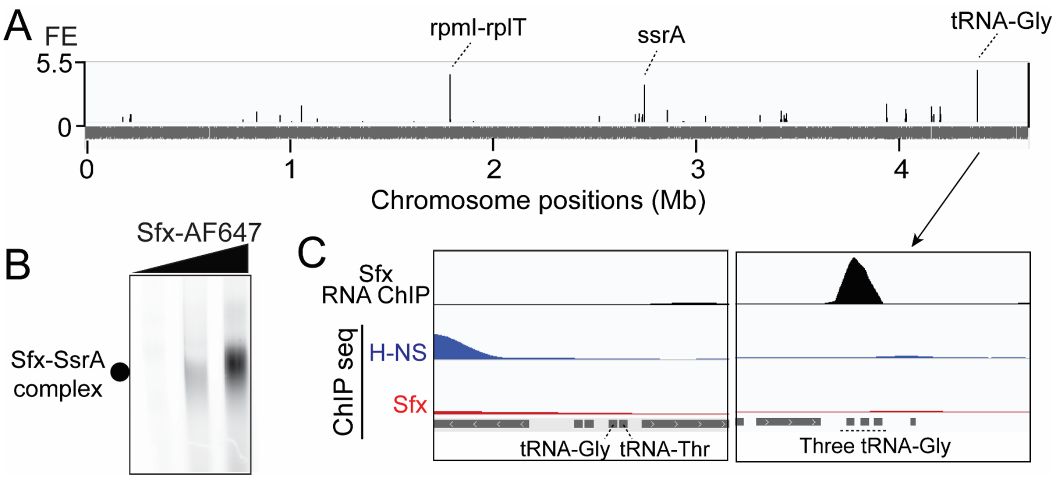

Supplementary Figure S6. RNA binding by Sfx. (**A**) RNA was ChIP-ed by Sfx-FLAG. Mean fold enrichment of two biological repeats is shown (FE = ChIP / Input). The top three enriched RNAs on the chromosome are indicated (**Dataset 1LM**); there was no enriched (FE > 2 in both biological repeats) RNA on R6K. (**B**) EMSA with 100 nM *ssrA* RNA and 1, 2, and 4 μM of Alexa Fluor 647 (AF647) labeled Sfx. The Sfx-SsrA complex is visualized by AF647 fluorescence. (**C**) Track views of significantly downregulated chromosomal genes upon the *sfx* deletion. FE of H-NS and Sfx ChIP-seq tracks ranged from 0 to 10, and FE of RNA ChIP-seq data ranged from 0 to 5.5. (Left) The downregulated tRNA-Gly (BW25113_RS20640) and tRNA-Thr (BW25113_RS20645) upon *sfx* deletion have no Sfx bound to their coding regions or the tRNAs themselves. (Right) In the RNA ChIP-seq data, we can see three enriched tRNA-Gly (BW25113_RS21635, BW25113_RS21640, BW25113_RS21645), but their RNA levels determined by RNA-seq are not changed.

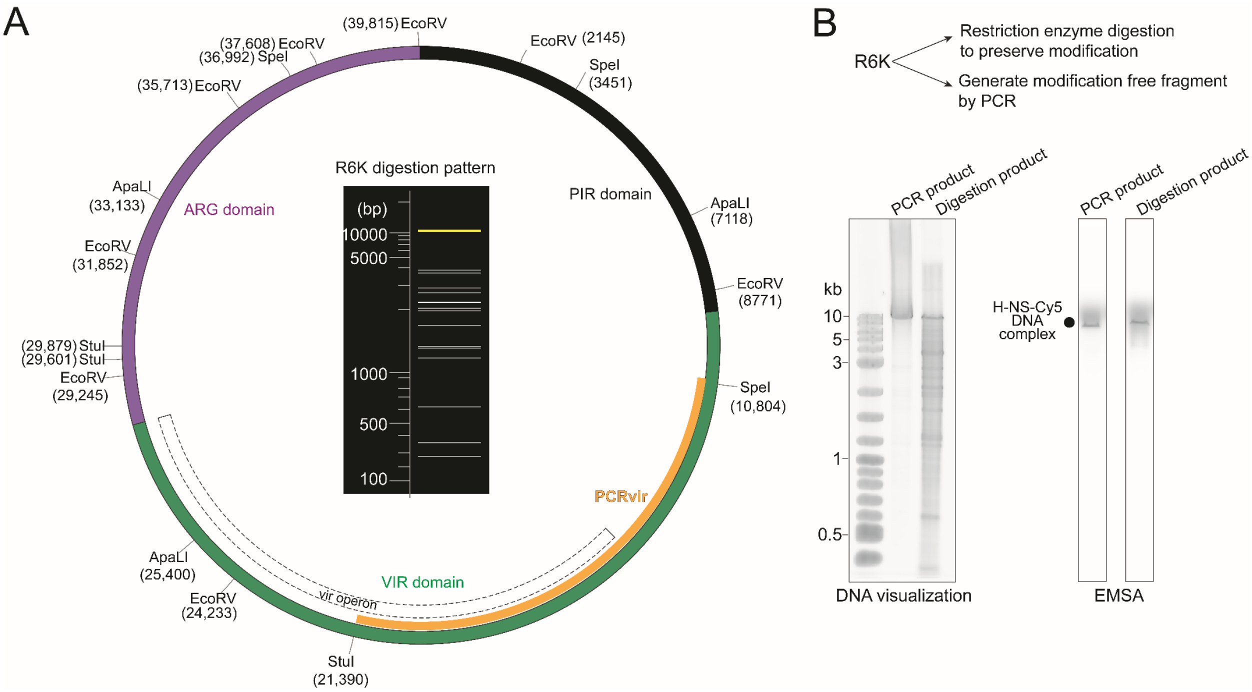

Supplementary Figure S7. DNA modification cannot explain the H-NS exclusion from the VIR domain. (**A**) R6K digestion map. The R6K digestion pattern is generated by NEBcutter v3.0.23. The R6K ARG, PIR, and VIR domains are colored in purple, black, and green, respectively. The tested DNA fragment (PCRvir) is indicated in orange. (**B**) DNA fragments containing the *vir* operon (PCRvir in panel **A**) were generated by PCR (no modification) or digestion with *Apa*LI, *Eco*RV, *Spe*I, and *Stu*I restriction enzymes (to preserve potential DNA modifications) and used for EMSA with Cy5-labeled H-NS. To visualize the DNA fragments, 300 ng of DNA were stained with SYBR Gold Nucleic Acid Gel Stain (ThermoFisher). The 10.6 kb PCRvir fragment has similar intensity, quantified with ImageJ, in both PCR and digestion products (Left). One µM of H-NS-Cy5 was incubated with 4 ng/µl PCR product or digestion product for 15 min at room temperature, and the reactions were analyzed on 1% agarose gel at 100 V in 0.5x TBE buffer. After running the gel for 2 h at 4 °C, the gel was imaged with Amersham Typhoon 5 (Right). Although many fragments are present in the digestion product, the H-NS-Cy5 + PCRvir fragment is the single strong band. The band intensity representing DNA-bound H-NS-Cy5 was quantified, and the ratio of band intensity in the PCR product to that in the digestion product is 1.14 ± 0.16 (Mean ± SD; n = 3).

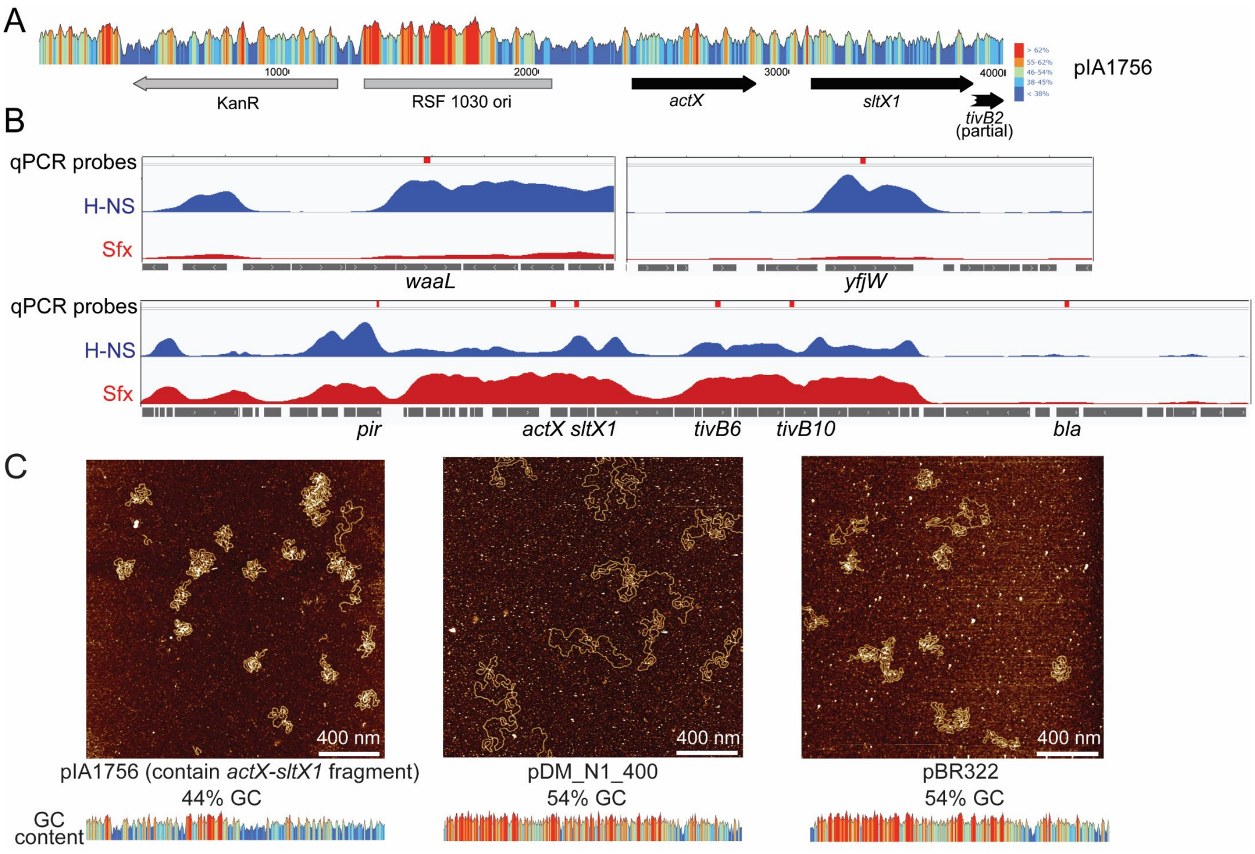

Supplementary Figure S8. Shuffling the *actX-sltX1* fragment. (**A**) plasmid map of pIA1756. The *actX-sltX1* transcription is under the control of Sfx. RT-qPCR shows 90 ± 9.9 (n = 3) folds of suppression in the presence of Sfx. GC content (calculated with Snapgene v8.2.2) is shown above the genes. (**B**) Positions of qPCR probes (red bars) are indicated above the ChIP-seq peaks. ChIP-seq tracks are FE ranging from 0 to 13. (**C**) AFM images of pIA1756, a plasmid with pBR322 backbone (pDM_N1_400), and the pBR322 plasmid. The scale bar is 400 nm. GC content distributions are shown below the images (same scale as panel **A**).

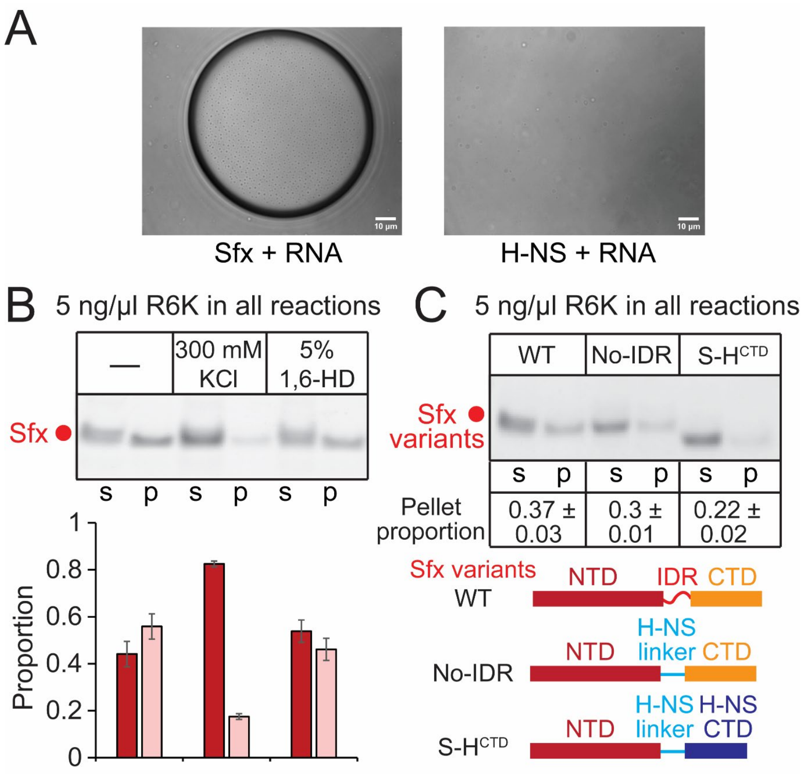

Supplementary Figure S9. Sfx phase separation is sensitive to salt concentration and is promoted by the Sfx IDR and CTD. (**A**) Bright field views of microscopy show that 5 µM Sfx forms droplets with 30 ng/µl total RNA extracted from *E. coli* in the presence of 8% PEG8000, but H-NS does not under the same condition. (**B**) Phase separation formation was challenged with 300 mM KCl or 5% (w/v) 1,6-Hexanediol (1,6-HD) in a pelleting assay. s, supernatant. p, pellet. Error bars represent SD (n = 3). (**C**) Three Sfx variants (1.5 µM each) indicated by the cartoon schematic were tested in a pelleting assay at 150 mM KCl. WT, wild-type. Data are presented as mean ± SD (n = 3). The pelleting assay was performed as in **Fig. 6B**.

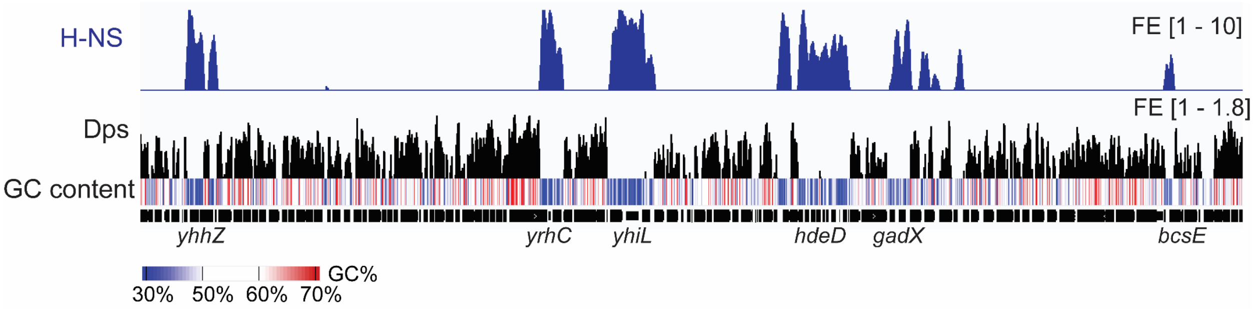

Supplementary Figure S10. ChIP-seq tracks of H-NS (this work) and Dps from the stationary phase (24-hour sample, see reference (7)). A 130 kbp region of the chromosome demonstrates that Dps binds to high GC regions and is excluded from H-NS binding regions. The tracks represent FE. FE ranges from 1 to 10 for the H-NS track, and from 1 to 1.8 for the Dps track. The GC content, calculated in a 25-bp sliding window, is shown as a heatmap.

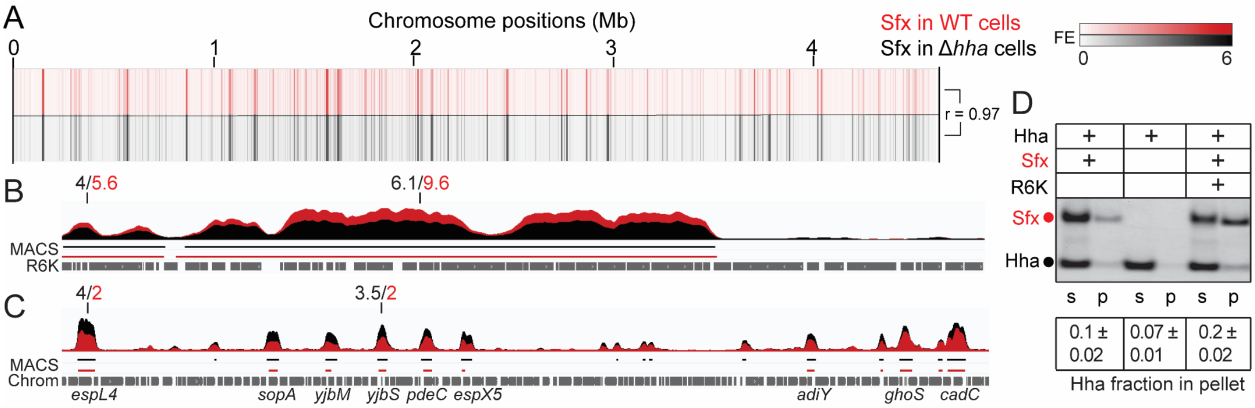

Supplementary Figure S11. ChIP-seq tracks of Sfx in wild-type (WT) and Δ*hha E. coli* cells. (**A**) Heatmap tracks on the chromosome. Spearman correlation (r) is calculated by binning reads at 300 bp. (**B**) The peak heights of Sfx on R6K are decreased in the absence of Hha. The peaks represent FE in the range of 0 to 11. Example peak heights are shown in panels B and C; black, Sfx in Δ*hha* cells; red, Sfx in WT cells. MACS plot highlights statistically significant bound regions. (**C**) A 169 kbp region of the chromosome (Chrom) shows that Sfx peaks after the *hha* deletion become higher, possibly reflecting the decreased binding of H-NS. The peaks represent FE in the range of 0 to 5. (**D**) A pelleting assay showing that Hha co-sediments with Sfx. In the reaction, 2 μM Sfx was mixed with 5 μM Hha and 5 ng/μl R6K, where indicated. After incubating for 10 min at room temperature (~23 °C), the reactions were spun at 21,000 x *g* for 25 min at 20 °C. s, supernatant. p, pellet. The Hha fraction in the pellet is presented as Mean ± SD (n = 3). Using band intensity, it's estimated that the molar ratio of Sfx/Hha in the pellet is 1, which is similar to the H-NS/Hha complex (8).

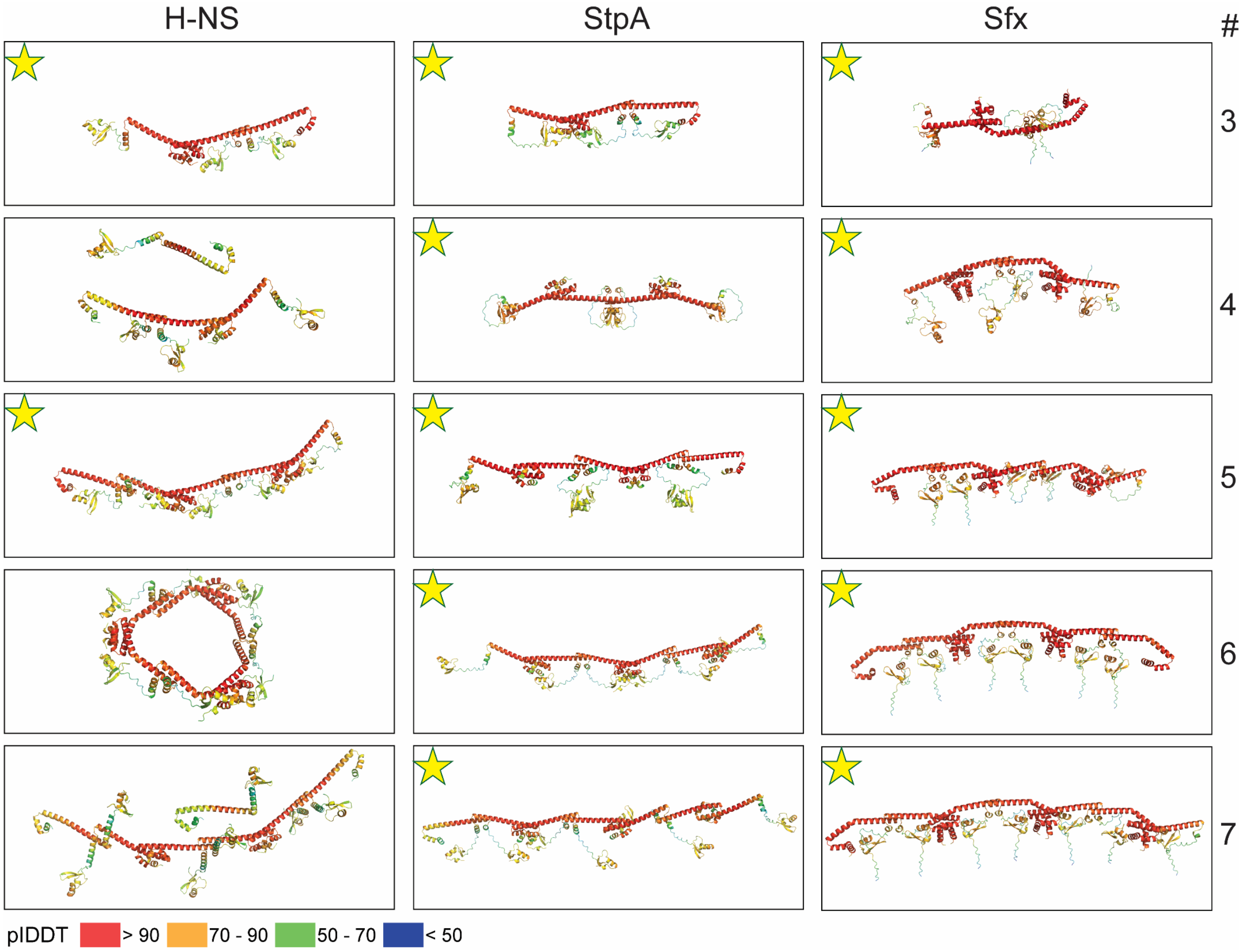

Supplementary Figure S12. Multimer prediction by AlphaFold3. The gold stars indicate the formation of undisrupted oligomers. The numbers on the right indicate the number of molecules used for prediction. The results show that Sfx and StpA can form oligomers in all conditions, while only two worked for H-NS.

Supplementary Table S1. Plasmids, strains, and primers. Lab collection IDs are assigned to all materials.

Plasmids	Description	Source
**pSC101 CmR plasmids for *in trans* expression of H-NS and Sfx from native promoters**		
p450	Empty pHSG576	(9)
p451	pHSG576-P*sfx*-*sfx*-FLAG	This work
p452	pHSG576-P*sfx*-*sfx*	(10)
p487	pHSG576-P*hns*-hns; pSC101 Cm^R	(10)
**pRSF KanR plasmids for protein overexpression; T7 promoter-His_{10}-SUMO**		
pIA1480	Wild type H-NS overexpression vector	This work
pIA1481	Wild type Hha overexpression vector	This work
pIA1649	Wild type Sfx overexpression vector	This work
pIA1701	Sfx with a unique C-terminal Cys	This work
pIA1720	H-NS with a unique C-terminal Cys	This work
pIA1747	Sfx variant (No-IDR) overexpression vector	This work
pIA1763	Sfx variant (S-H^{CTD}) overexpression vector	This work
**R6K derivative plasmids; AmpR**		
p103	R6K wild type	(10)
pIA1548	R6KΔ*sfx*	This work
pIA1682	R6K75; the transposon region is removed except for the *bla* gene	This work
pIA1698	R6K75Δ*sfx*	This work
pIA1710	R6KΔ*sfx* with *yfjW* gene inserted into the ARG domain	This work
pIA1721	R6K75*sfx*-FLAG; FLAG tag is added to the C-terminus of Sfx	This work
pIA1767	P*actX*-*yfp* inserted in R6KΔ*sfx*	This work
pIA1768	P*actX*-*yfp* inserted in R6K	This work
**Miscellaneous plasmids**		
p28	pBAD33 P15A Cm^R	(11)
pDM-N1-400	5848 bp pBR322 backbone vector (Addgene #170474)	(12)
pIA1723	P*actX*-*yfp* reporter; P*actX* cloned into pYPet-His (Addgene #14031)	This work
pIA1756	*actX*-*sltX1*-*tivB2* fragment cloned into a pRSF plasmid	This work
**Strains**	**Genotype**	**Source**
IA294	*Escherichia coli* K-12 BW25113 Keio collection wild type	(13)
IA357	*E. coli* BW25113 Δ*hha*::Kan^R	(13)
IA428	*E. coli* BW25113 *lysA*::Kan^R	(13)
IA765	*Escherichia coli* K-12 MG1655	Lydia Freddolino
IA969	*E. coli* MG1655 *hns*-3xFLAG	Joseph Wade
IA1030	*E. coli* BW25113 *argE*::Tet^R	This work
BW55	*E. coli* MG1655 *yfjw*::*actX*-*sltX1::*Kan^R	This work
BW56	*E. coli* MG1655 *hns*-3xFLAG *yfjw*::*actX*-*sltX1*::Kan^R	This work
**Primers**	**Sequences (5' – 3')**	**Usage**

3044	CTTGATCGTTGGGAACCGGAG	*bla* qPCR
3045	TATCCGCCTCCATCCAGTCT	
3063	TCCTCCTTCTGGCTATCGGT	*tivB10* qPCR
3064	TGTCATCCCTCTGGCTCTGA	
3069	CGAAAGGAGGGCGATAGTGG	*sltX1* qPCR
3070	TTGTGCATGGCGAGAAAAGC	
3086	CCGGGGGTATTGTTGCTCTT	*tivB6* qPCR
3087	AATGCCAACGCACCGAAAAG	
3079	CTGAAGCAGCACGCAAAGAG	*rho* qPCR
3080	GCGCAAAATAGCGTTCACCT	
3113	CCACGACAAGATTTGCAGCC	*actX* qPCR
3114	GAAGCCCGGTGATAGTGGTC	
3325	TCAACAGCATCTACATGATGGCC	*rpoC* qPCR
3326	CGCCATCAGACCACGCATAC	
3335	GCAAGCGATGGGGTGATTTT	*pir* qPCR
3336	ATATGGCGCTTGCTCCCATT	
3343	GGCGTGTTACGGTGAAAACC	*cat* qPCR
3344	AAACTCACCCAGGGATTGGC	
3490	GGAGCATATGGCCTCGACTC	*ihfB* qPCR
3491	TCGCCAGTCTTCGGATTACG	
3552	CAGACACACCAACGTATCTGGA	*yfjW* qPCR
3553	GGAAGGGCATAACAACCGGA	
3554	GGATAGTTAGTGGCGTTGCG	*waaL* qPCR
3555	GGGAACAGGAGTAGGGTTGC	
2282	GCTAGCACAAGGGAGTAGTTG	*actX-sltX1* EMSA fragment
2771	AGCAAGATTTGTGCATGGCG	
2659	CTAAAACAGTCGAAGTAACACC	PCR *vir* EMSA fragment
3060	CCCCGGCATTTTTCCCCATA	
3465	CAGTAATACGACTCACTATAGGGGCTGATTCTGGATTCGA	T7 promoter-*ssrA* template for making SsrA RNA
3466	TTTGGTGGAGCTGGCGGGAG	
3643	AGATGTGTAACCAGTCGCTGA	Bridging assay template
3644	CTGGTCAGTGCCCAAATGAG	Use with 3643 to make DNA probe
3645	[BTN]CAACAGCGGGAAAAAGTCGTC	Use with 3643 to make DNA bait

Supplementary References

1. Lartigue, M.F., Leflon-Guibout, V., Poirel, L., Nordmann, P. and Nicolas-Chanoine, M.H. (2002) Promoters P3, Pa/Pb, P4, and P5 upstream from bla(TEM) genes and their relationship to beta-lactam resistance. *Antimicrob Agents Chemother*, **46**, 4035-4037.
2. Reese, M.G. (2001) Application of a time-delay neural network to promoter annotation in the Drosophila melanogaster genome. *Comput Chem*, **26**, 51-56.
3. Shen, B.A., Hustmyer, C.M., Roston, D., Wolfe, M.B. and Landick, R. (2022) Bacterial H-NS contacts DNA at the same irregularly spaced sites in both bridged and hemi-sequestered linear filaments. *iScience*, **25**.
4. van der Valk, R.A., van Erp, B., Qin, L., Moolenaar, G.F. and Dame, R.T. (2024) Quantitative Determination of DNA Bridging Efficiency of Chromatin Proteins. *Methods Mol Biol*, **2819**, 443-454.
5. Chandraprakash, D. and Seshasayee, A.S. (2014) Inhibition of factor-dependent transcription termination in Escherichia coli might relieve xenogene silencing by abrogating H-NS-DNA interactions in vivo. *J Biosci*, **39**, 53-61.
6. Gavrilov, A.A., Shamovsky, I., Zhegalova, I., Proshkin, S., Shamovsky, Y., Evko, G., Epshtein, V., Rasouly, A., Blavatnik, A., Lahiri, S. *et al.* (2025) Elementary 3D organization of active and silenced E. coli genome. *Nature*, **645**, 1060-1070.
7. McCarthy, L.A., Way, L.E., Dai, X., Ren, Z., Fuller, D.E.H., Dhiman, I., Larkin, L., Sieben, J.J.D., Westerlaken, I., Abbondanzieri, E.A. *et al.* (2026) Dps binds and protects DNA in starved Escherichia coli with minimal effect on chromosome accessibility, dynamics, and organization. *Nucleic Acids Research*, **54**.
8. Ali, S.S., Whitney, J.C., Stevenson, J., Robinson, H., Howell, P.L. and Navarre, W.W. (2013) Structural insights into the regulation of foreign genes in Salmonella by the Hha/H-NS complex. *J Biol Chem*, **288**, 13356-13369.
9. Takeshita, S., Sato, M., Toba, M., Masahashi, W. and Hashimoto-Gotoh, T. (1987) High-copy-number and low-copy-number plasmid vectors for lacZ alpha-complementation and chloramphenicol- or kanamycin-resistance selection. *Gene*, **61**, 63-74.
10. Wang, A., Cordova, M. and Navarre, W.W. (2025) Evolutionary and functional divergence of Sfx, a plasmid-encoded H-NS homolog, underlies the regulation of IncX plasmid conjugation. *mBio*, **16**, e02089-02024.
11. Guzman, L.M., Belin, D., Carson, M.J. and Beckwith, J. (1995) Tight regulation, modulation, and high-level expression by vectors containing the arabinose PBAD promoter. *J Bacteriol*, **177**, 4121-4130.
12. Xu, W., Yan, Y., Artsimovitch, I., Dunlap, D. and Finzi, L. (2022) Positive supercoiling favors transcription elongation through lac repressor-mediated DNA loops. *Nucleic Acids Res*, **50**, 2826-2835.
13. Baba, T., Ara, T., Hasegawa, M., Takai, Y., Okumura, Y., Baba, M., Datsenko, K.A., Tomita, M., Wanner, B.L. and Mori, H. (2006) Construction of Escherichia coli K-12 in-frame, single-gene knockout mutants: the Keio collection. *Mol Syst Biol*, **2**, 2006.0008.
